# Aquaculture Soft Coral *Lobophytum crassum* as a Producer of Anti-Proliferative Cembranoids

**DOI:** 10.3390/md16010015

**Published:** 2018-01-07

**Authors:** Bo-Rong Peng, Mei-Chin Lu, Mohamed El-Shazly, Shwu-Li Wu, Kuei-Hung Lai, Jui-Hsin Su

**Affiliations:** 1 Doctoral Degree Program in Marine Biotechnology, National Sun Yat-Sen University (NSYSU), 70 Lien-Hai Road, Kaohsiung 80424, Taiwan; pengpojung@gmail.com; 2 Doctoral Degree Program in Marine Biotechnology, Academia Sinica, 128 Academia Road, Section 2, Nankang, Taipei 11529, Taiwan; 3 National Museum of Marine Biology & Aquarium, Pingtung 94450, Taiwan; jinx6609@nmmba.gov.tw; 4 Graduate Institute of Marine Biology, National Dong Hwa University, Pingtung 94450, Taiwan; 5 Department of Pharmacognosy and Natural Products Chemistry, Faculty of Pharmacy, Ain-Shams University, Organization of African Unity Street, Abassia, Cairo 11566, Egypt; mohamed.elshazly@pharma.asu.edu.eg; 6 Department of Pharmaceutical Biology, Faculty of Pharmacy and Biotechnology, German University in Cairo, Cairo 11432, Egypt; 7 General Study Center, National Kaohsiung Marine University, Kaohsiung 80543, Taiwan; wusl@webmail.nkmu.edu.tw

**Keywords:** aquaculture *Lobophytum crassum*, cembranoids, Molt 4 leukemia, anti-proliferation, SAR

## Abstract

Our continuous search for marine bioactive secondary metabolites led to the screening of crude extracts from a variety of aquaculture soft corals. The ethyl acetate (EtOAc) extract of *Lobophytum crassum* showed a distinctive chemical profile that was different from the wild type. It demonstrated significant anti-proliferative activity against Molt 4 leukemia cell with an IC_50_ value of 1 μg/mL after 24 h. Chemical investigation focusing on the unique peaks in *L. crassum* profile led to the discovery of a new α-tocopherol crassumtocopherol C (**1**), and two new cembrane-based diterpenoids culobophylins D (**2**) and E (**3**), along with ten known cembranoids (**4**–**13**). The structures of these isolates were elucidated using extensive spectroscopic techniques and a comparison with previously published data of related metabolites. Compound **2** was found to possess the first identified saturated internal C_4_-O-C_14_ linkage six-membered ring among all cembrane-type diterpenoids. The anti-proliferative activity of all the isolates (except **3**) was evaluated against a limited panel of leukemia cell lines (Molt 4, K562, U937, and Sup-T1). The major compounds **8** and **10** exhibited the most anti-proliferative potent effect, with IC_50_ values ranging from 1.2 to 7.1 μM. The Structure Activity Relationship (SAR) of the isolates suggested that the presence of lactone moieties is crucial for the anti-proliferative activity against leukemia cells. Our work indicated that the development of an efficient aquaculture protocols for soft corals led to the discovery of new secondary metabolites with unique structural features. Such protocols can lead to a sustainable supply of biologically active compounds in enough quantities for the pharmaceutical industry.

## 1. Introduction

In recent years, aquaculture techniques of soft corals witnessed significant advancements in terms of conditions and productivity. Researchers have been able to obtain larger amounts of soft corals, and thus larger amounts of bioactive metabolites enabling them to investigate their biological activities in many pharmacological assays and even producing enough quantities for clinical trials [1]. In the past few decades, a variety of aquaculture protocols for soft corals were established and the produced soft corals were extensively studied in terms of their chemical profiles and pharmacological properties. For example, the soft coral *Sinularia flexibilis* was cultured by our institute and the anti-neuroinflammatory and analgesic activities of its active constituent, flexibilide, were studied. The other active compound, 11-*epi*-sinulariolide acetate, was examined for its potential anti-cell migration and invasion effects on hepatocellular carcinoma cells [2,3]. 13-Acetoxysarcocrassolide, a bioactive compound that was isolated from the cultured soft coral *Sarcophyton crassocaule*, was found to possess anti-proliferative and apoptosis-inducing activities against AGS cells (human gastric adenocarcinoma) [4]. Excavatolide B, a bioactive compound isolated from the cultured soft coral *Briareum excavatum* inhibited mRNA expression of the proinflammatory mediators, inducible nitric oxide synthase (iNOS) and cyclooxygenase-2 (COX-2), in lipopolysaccharide (LPS)-challenged murine macrophages [5]. Through the application of aquiculture techniques, these compounds could be adequately produced for pharmacological studies and clinical trials by health authorities and pharmaceutical companies.

In our continuous search for bioactive agents from soft corals, the anti-proliferative activity of six cultured samples *Briareum excavatum*, *Lobophytum crassum*, *Paralemnalia thyrsoides*, *Sarcophyton* sp., *Sinularia flexibilis,* and *Sinularia sandensis*, were examined. Among the evaluated extracts, the ethyl acetate extract of *Lobophytum crassum* exhibited potent anti-proliferative activity against Molt-4 (human acute lymphoblastic leukemia) cancer cells, with an IC_50_ value of 1 μg/mL after 24 h. The chemical profiles of the aquaculture and wild *L. crassum* extracts using high performance liquid chromatography (HPLC) suggested a considerable difference between their chemical contents. Based on these results, we investigated the chemical constituents of the aquaculture and wild *L. crassum* soft corals and evaluated their anti-proliferative activity. A new α-tocopherol (**1**) and two new cembranoids (**2** and **3**), along with ten known cembranoids (**4**–**13**) were isolated. The anti-proliferative effect of all the isolates (except **3**) against four leukemia cell lines (K562, Molt-4, U937, and Sup-T1) was evaluated.

## 2. Results

### 2.1. Chemical Profile of Soft Coral Lobophytum crassum from Various Sources

**Figure 1:**
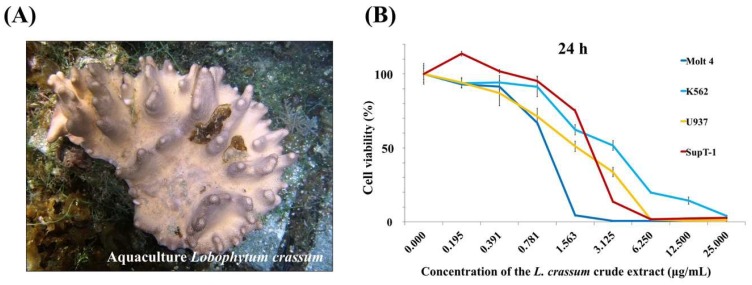
(**A**) Aquatic ecology of aquaculture *L. crassum* and (**B**) the effect of its ethyl acetate (EtOAc) extract on cell viability of leukemia cancer cell lines after 24 h.

**Figure 2:**
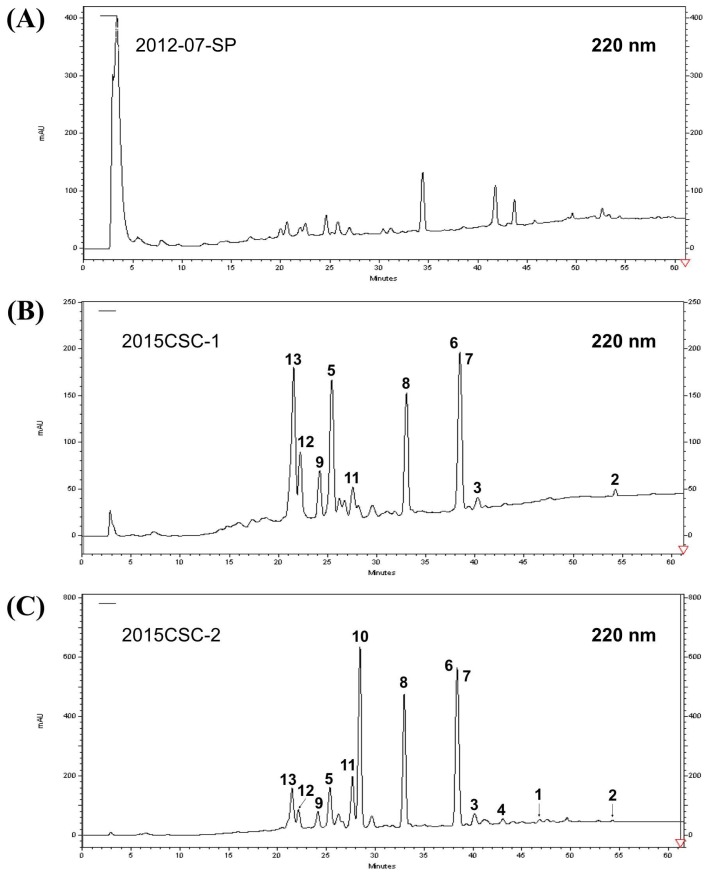
High performance liquid chromatography (HPLC) chromatograms of EtOAc extracts of (**A**) wild *L. crassum* collected in Pingtung (specimen No. 2012-07-SP), and two batches of aquaculture *L. crassum* soft corals (**B**) 2015CSC-1 and (**C**) 2015CSC-2.

The anti-proliferative activity of the ethyl acetate (EtOAc) extracts of six cultured soft corals maintained at the National Museum of Marine Biology & Aquarium, Pingtung, Taiwan was examined and the aquaculture soft corals (batch no. 2015CSC-2) (Figure 1A) was selected for further study since it exhibited the most potent effect against several leukemia cells (Figure 1B). To determine the differences in secondary metabolites content between the wild and aquaculture soft corals, HPLC qualitative analysis was performed. The HPLC chromatogram (Figure 2) of the two batches (2015CSC-1 and 2015CSC-2) from aquaculture *L. crassum* EtOAc extracts displayed a similar chemical profile, but they were different from the wild sample. The distinct peaks in the aquaculture soft coral *L. crassum* EtOAc extract were isolated and identified to trace the anti-proliferative components.

### 2.2. Chemical Identification of Characteristic Cembranoids

**Figure 3:**
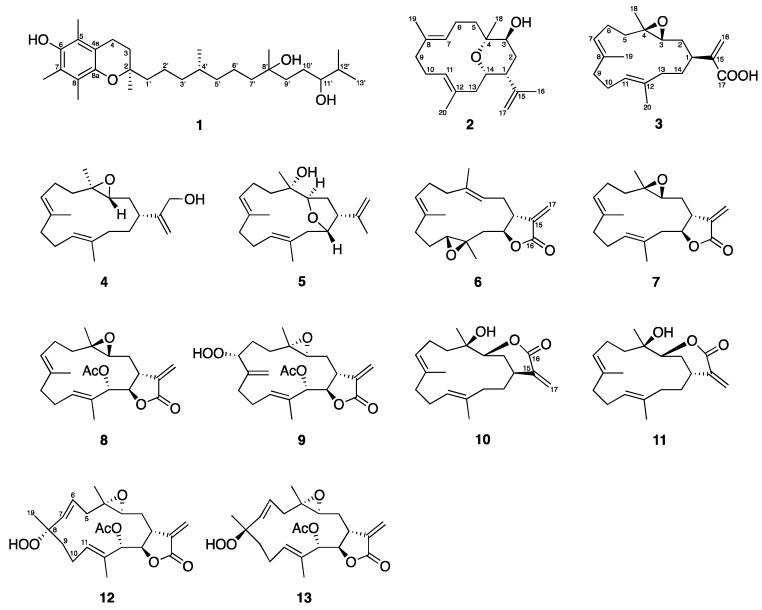
Tocopherol and cembranoids isolated from the aquaculture soft coral of *L. crassum*.

The freeze-dried specimen of the aquaculture soft coral *L. crassum* (2015CSC-2) was extracted exhaustively with EtOAc, and the obtained crude extract was further fractionated and purified using normal and reversed phase column chromatography based on the unique peaks from the HPLC profile. Three new compounds were isolated, including crassumtocopherol C (**1**), culobophylin D (**2**) and E (**3**), along with ten known cembranoids (**4**–**13**) identified as lobocrassin C (**4**) [6], lobophylin (**5**) [7], crassocolide E (**6**) [8], sarcocrassocolide (**7**) [9], 13-acetoxysarcocrassocolide (**8**) [9], sarcocrassocolide M (**9**) [10], 14-deoxycrassin (**10**) [11], lobocrassin B (**11**) [6], sarcocrassocolide F (**12**) [12], and sarcocrassocolide G (**13**) [12] (Figure 3).

**Figure 4:**
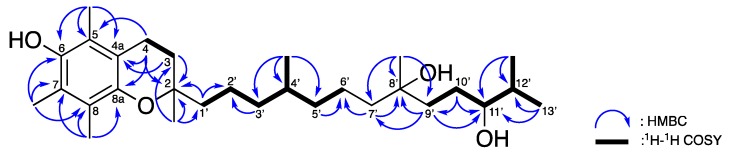
Selective ^1^H–^1^H COSY and HMBC correlations of **1**.

Compound **1** was isolated as a yellow oil. The high-resolution mass spectroscopy and the ^13^C NMR data suggested a molecular formula of C_29_H_50_O_4_ (five degrees of unsaturation) by observing a pseudo-molecular ion peak at *m*/*z* 485.3599 ± 0.0005 [M + Na]^+^. The ^1^H, ^13^C, and HSQC NMR (Table 1) revealed the presence of a characteristic hexa-substituted aromatic moiety deduced from the four olefinic quaternary carbons [*δ*_C_ 117.4 (C-4a), 118.7 (C-5), 121.2 (C-8), and 122.6 (C-7)], and two oxy olefinic quaternary carbons [144.5 (C-6) and 145.5 (C-8a)]. The HMBC spectroscopic cross-peaks (Figure 4) from the two methylene protons [*δ*_H_ 1.79 (H-3, m) and 2.61 (H-4, t, 7.0)] to C-4a; from H-3 to an sp^3^ oxy quaternary carbon *δ*_C_ 74.4 (C-2); and from H-4 to C-8a further constructed a 6-hydroxy-5,7,8-trimethylchroman moiety. A 2,6,10-trimethyltridecane-3,6-diol was identified based on 1D (Table 1) and 2D NMR data (Figure 4) which sorted out the four methyl groups [*δ*_C_/*δ*_H_ 17.3 (C-12’-Me)/0.93 (H-12’-Me, d, *J* = 7.0 Hz), 19.6 (C-4’-Me)/0.86 (H-4’-Me, d, *J* = 6.5 Hz), 19.8 (C-13’)/0.93 (H-13’, d, *J* = 7.0 Hz), and 26.7 (C-8’-Me)/1.17 (H-8’-Me, s)], eight methylenes [*δ*_C_/*δ*_H_ 20.8 (C-2’)/1.40, 1.47 (H-2’, m), 21.3 (C-6’)/1.26 (H-6’, m), 28.2 (C-10’)/1.48, 1.56 (H-10’, m), 37.3 (C-3’)/1.09, 1.26 (H-3’, m), 37.3 (C-5’)/1.14, 1.26 (H-5’, m), 38.2 (C-9’)/1.40 (H-9’, m), 39.2 (C-1’)/1.47, 1.56 (H-1’, m), and 42.8 (C-7’)/1.52, 1.65 (H-7’, m)], three methines [*δ*_C_/*δ*_H_ 32.4 (C-4’)/1.42 (H-4’, m), 33.7 (C-12’)/1.69 (H-12’, m), 77.4 (C-11’)/3.36 (H-11’, m)], and one oxy quaternary carbon [*δ*_C_ 72.6 (C-8’)]. The HMBC correlations (Figure 4) from H-1’, H-3, and H-4 to C-2; from an additional methyl [*δ*_H_ 1.24 (H-2-Me, s)] proton to C-2, C-3, and C-1’; connected these two subunits and indicated an α-tocopherol skeleton.

The assignment of the relative configurations of the atoms attached to C-2, C-4’, and C-8’ was determined by the ^13^C NMR (Table 1) chemical shifts (measured in CDCl_3_) when compared to the previously reported data of crassumtocopherol A [13]. The identical ^13^C chemical shifts (except for C-10’–C-13’) between **1** and crassumtocopherol A suggested a similar relative configuration of 2R*, 4’R*, which were consistent with those of the naturally occurring *α*-tocopherol [14]. Thus, the structure of **1** was established and named as crassumtocopherol C.

**Figure 5:**
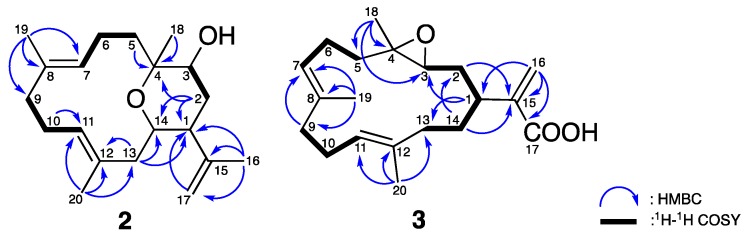
Selective ^1^H–^1^H COSY and HMBC correlations of **2** and **3**.

Compound **2**, a white amorphous powder, was found to possess a molecular formula of C_20_H_32_O_2_ based on the ^13^C NMR and quasi-molecular ion peak in the HRESIMS, indicating 5 degrees of unsaturation. The IR spectra suggested the presence of OH (3299 cm^−1^) and C=C (1642 cm^−1^) functionalities. The planar structure of **2** was elucidated based on one-dimensional (1D) and two-dimensional (2D) NMR spectroscopic data, ^1^H–^1^H COSY and HMBC experiments. The 1D NMR data (Table 2) combined with the HSQC spectroscopic demonstrated the existence of two pairs of C=C double bonds [*δ*_C_/*δ*_H_ 130.8 (C-12), 129.7 (C-7)/5.01 (H-7, br d, *J* = 11.0 Hz), 128.4 (C-8), 128.0 (C-11)/4.87 (H-11, br s)], one terminal double bonds [*δ*_C_/*δ*_H_ 145.3 (C-15), 113.7 (C-17)/4.88 (H-17, br s), 4.97 (H-17, d, *J* = 1.5 Hz)], one sp^3^ oxy quaternary carbon [*δ*_C_ 76.9 (C-4)], two oxymethines [*δ*_C_/*δ*_H_ 71.2 (C-3)/3.72 (H-3, dd, *J* = 11.0, 6.0 Hz), 66.5 (C-14)/3.61 (H-14, dt, *J* = 11.5, 3.0 Hz), and four tertiary methyls [*δ*_H_ 1.81 (H-16, s), 1.56 (H-19, s), 1.50 (H-20, s), and 1.08 (H-18, s)]. From the COSY spectra (Figure 5), four partial structures of consecutive proton spin systems were constructed extending from H-2 to H-3, from H-5 to H-7, and from H-9 to H-11. The connection between each partial structure was further supported by the HMBC correlations (Figure 5) from H-2, H-5, and H-18 to C-4; from H-19 to C-7, C-8, and C-9; from H-20 to C-11, C-12, and C-13; from H-2, H-13, H-16, and H-17 to C-1; and from H-2 and H-13 to C-14, which established a basic type of cembranoid skeleton (a three-methyl substituted 14-membered ring system) [15].

**Figure 6:**
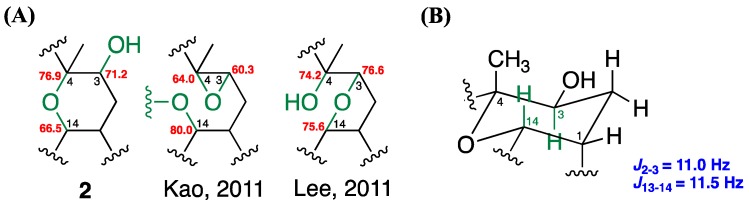
Determination of the suitable ether bridges in **2** by (**A**) comparison of ^13^C NMR chemical shifts, and (**B**) the confirmation of the coupling constants of axial protons in C_4_-O-C_14_ linkage six-membered ring system.

**Figure 7:**
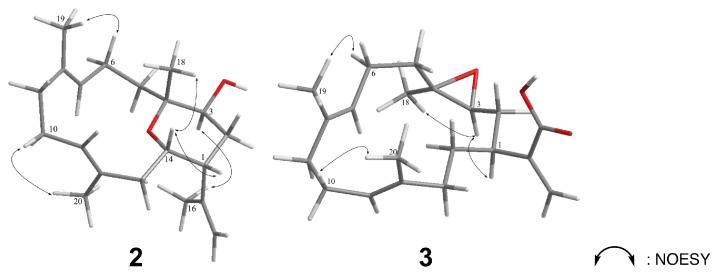
Selective NOESY correlations of **2** and **3**.

The careful analysis of these data established the basic structure of **2**, suggesting the presence of three olefins and one ring system, accounting for four of the total five degrees of unsaturation. The remaining one degree of unsaturation was attributed to an additional cyclic structure in **2**. According to the NMR and MS data, there were three oxygenated carbons [*δ*_C_/*δ*_H_ 71.2 (C-3)/3.72 (H-3, dd, *J* = 11.0, 6.0 Hz), 76.9 (C-4), and 66.5 (C-14)/3.61 (H-14, dt, *J* = 11.5, 3.0 Hz)], bearing only two oxygen atoms, which suggested an ether bridge either between C-3 and C-4, C-3 and C-14, or C-4 and C-14. By comparing the ^13^C chemical shifts with those of the previously reported compounds (Figure 6A), the C_4_-O-C_14_ linkage was deduced to be the most suitable structure instead of C_3_-O-C_4_ [*δ*_C_ 60.3 (C-3), 64.0 (C-4), 80.0 (C-14)] [6], and C_3_-O-C_14_ [*δ*_C_ 76.6 (C-3), 74.2 (C-4), 75.6 (C-14)] [16]. This hypothesis was further supported by the detection of large proton coupling constants at H-3 (*J* = 11.0 Hz) and H-14 (*J* = 11.5 Hz), which indicated an axial orientation of these two protons in a saturated six-membered ring system (Figure 6B) [17]. The β-H-1 (equatorial), α-H-3 (axial), β-Me-18 (axial), and β-H-14 (axial) orientations were also suggested. These suggestions regarding the protons and methyl orientations were consistent with those that were determined by the NOESY spectra [between H-14/H-18, H-1/H-14, and H-3/H-16] (Figure 7). Moreover, the NOESY correlations between H-6/H-19 and H-10/H-20, as well as the missing NOESY correlations between H-11/H-20 and H-7/H-19 suggested *E*-configurations for these two C=C double bonds [18]. Accordingly, the relative configuration of **2** was suggested to be 1R*, 3S*, 4S*, 14S*, 7*E*, and 11*E,* and the compound was named culobophylin D.

Compound **3**, a white amorphous powder, gave a pseudo molecular ion peak, and the HRESIMS and ^13^C NMR analysis proposed a molecular formula of C_20_H_30_O_3_, with six degrees of unsaturation. The IR spectra showed absorptions of a hydroxy (3417 cm^−1^) and unsaturated carbonyl (1693 cm^−1^), as well as C=C (1621 cm^−1^) functional groups. The ^1^H, ^13^C (Table 3), DEPT, and HSQC NMR spectra suggested a carbonyl group [*δ*_C_ 170.2 (C-17)], three pairs of C=C double bonds [*δ*_C_/*δ*_H_ 144.1 (C-15), 135.2 (C-12), 133.2 (C-8), 126.3 (C-16)/6.36 (H-16, d, *J* = 1.0 Hz), 5.65 (H-16, s), 124.8 (C-7)/5.11 (H-7, t, *J* = 6.5 Hz), and 123.8 (C-11)/5.16 (H-11, t, *J* = 6.5 Hz)], one sp^3^ oxygenated carbon [*δ*_C_ 60.9 (C-4)], one oxymethine [*δ*_C_/*δ*_H_ 62.9 (C-3)/2.84 (H-3, dd, *J* = 9.5, 3.0 Hz)], two tertiary methyls [*δ*_C_/*δ*_H_ 16.9 (C-19)/1.58 (H-19, s), and 15.6 (C-20)/1.62 (H-20, s)]. Detailed analysis of the above NMR data suggested that **3** possessed the same basic type cembrane-type diterpenoid skeleton as **2** [15]. It also differed from the previously reported pseudoplexauric acid methyl ester, in which the methyl ester was replaced with a carboxylic acid functionality [11]. The proposed structure was further confirmed by 2D NMR spectra, especially the ^1^H–^1^H COSY and HMBC spectroscopic data (Figure 5). Furthermore, the C_3_-O-C_4_ epoxide moiety was deduced by the characteristic absorption peak at 950 cm^−1^ in the IR spectra, as well as the ^13^C chemical shifts when compared with the previously reported data [**3**: *δ*_C_ 60.9 (C-3), 62.9 (C-4); Kao, 2011 [6]: *δ*_C_ 60.3 (C-3), 64.0 (C-4)] (Figure 6A).

The orientation of H-1 (*δ*_H_ 2.77 m) was assigned as α in compound **3** by comparing its data with those of pseudoplexauric acid [11]. The relative configuration was determined by analyzing the NOESY spectra. Since H-3 was correlated to H-1 and H-18, the α-orientations of both H-3 and H-18 were suggested. The large coupling constant (9.5 Hz) existed between H-2 and H-3 also confirmed the proposed conformation [17]. In addition, the *E* configuration of C-7/C-8 and C-11/C-12 C=C double bond systems was suggested by tracing NOESY cross-peaks between H-6/H-19 and H-10/H-20. Hence, the relative configuration was established as 1R*, 3S*, 4R*, 7*E*, and 11*E*, and the structure of compound **2** was elucidated and named culobophylin E. The structure of **3** was previously reported in a saponifying synthetic reaction that was derived from pseudoplexauric acid methyl ester, but without any data on the synthetic procedures, isolation, and identification [11].

Detialed NMR and spectrum data can be found in Supplementay Materials (Figures S1–S27).

### 2.3. Anti-Proliferative Activity of Isolated Cembranoids

Based on the anti-proliferative properties that were demonstrated by the EtOAc extract of aquaculture soft coral *L. crassum*, all of the isolated tocopherol and cembranoids (except **3**) were evaluated for their anti-proliferative activity against four leukemia cell lines (Molt 4, K562, U937, and Sup-T1) (Table 4). Compounds **6**–**10**, the cembranoids possessing α-methylene-*γ*-lactone or α-methylene-*δ*-lactone moieties, showed potent anti-proliferative activity. Compounds **8**, **10**, and **11** were the most active isolates. Since compounds **8** and **10** were the major components according to HPLC quantitative analysis, it was suggested that the anti-proliferative activity of *L*. *crassum* extract was attributed to these two cembranoids, and that the α-methylene-γ-lactone or α-methylene-*δ*-lactone moieties functionalities played a significant role in their activity against leukemia cancer cell lines.

## 3. Material and Methods

### 3.1. General Experimental Procedures

High resolution electrospray ionization mass spectrometry (HRESIMS) analyses were carried out on a Bruker APEX II instrument (Bruker Daltonik, Bremen, Germany). UV spectra were measured using JASCO V-650 ultraviolet spectrophotometers (JASCO, Tokyo, Japan). Infrared (IR) spectra were performed on a Fourier-transform IR spectrophotometer Varian Digilab FTS 1000 (Varian Inc., Palo Alto, CA, USA). NMR spectra were detected on a Varian Unity INOVA 500 FT-NMR instrument (Varian Inc., Palo Alto, CA, USA). Normal phase column chromatography was performed with 230−400 mesh silica gel (Merck, Darmstadt, Germany). TLC was performed on 0.25 mm thick precoated Kieselgel 60 F_254_ (Merck, Darmstadt, Germany) and/or 0.25 mm RP-18 F_254S_ (Merck, Darmstadt, Germany) coated plates, and then visualized by immersing with 10% H_2_SO_4_ and heating on a hot plate. Hitachi L-2130 and L-7100 pumps, Rheodyne 7725 injection port and a Hitachi L-2455 Photodiode Array Detector (Hitachi, Tokyo, Japan), along with a preparative normal phase column Supelco Ascentis^®^ Si (10 mm × 250 mm) (supplied by Sigma-Aldrich, St. Louis, MO, USA) and a reversed phase column Supelco Ascentis^®^ C-18 (10 mm × 250 mm, C_18_) were used for reverse phase high performance liquid chromatography (RP-HPLC). All of the methods were carried out in accordance with the relevant guidelines and regulations.

### 3.2. Animal Material from Diverse Sources

Specimens of wild soft coral of *L. crassum* was originally collected by scuba diving from the coast of Pingtung, Taiwan, in 2015 (specimen No. 2012-07-SP). These corals were preserved and aquacultured in National Museum of Marine Biology & Aquarium (Pingtung, Taiwan). The aquaculture protocol was described below: For the domestication and culture development, the collected wild corals were cut into a few 4 to 5 cm of the sub-strains, these sub-strains then placed and attached naturally on the porous tile for breeding. These soft corals were reared in a seawater cylinder (four tons) with a cooler for control temperature (25–28 °C), and a LED coral lamp for 9–12 h light support per day. The ecological environment was arranged with live sea sands, live sea rocks, *Paracanthurus hepatus* fishes, snails, sea urchins, sea cucumbers, and other aquaculture soft corals of *Briareum* sp*.*, *Paralemnalia* sp*.*, *Sarcophyton* sp., and *Sinularia* sp. The aquaculture *L*. *crassum* soft corals were harvested in 2015, resulting in two batches (2015CSC-1 and 2015CSC-2).

### 3.3. HPLC Qualitative Analysis of L. crassum from Diverse Sources

Sample analysis of *L*. *crassum* from various sources was carried out on a Hitachi Elite LaChrom HPLC system (Hitachi, Tokyo, Japan) consisting of a Hitachi L-2130 pump, a Hitachi L-2455 Photodiode Array Detector. Liquid chromatography was performed using a BIOSIL Aqu-ODS-W-5u column (4.6 mm × 250 mm) (Bio-Rad, Hercules, CA, USA). The mobile phase was a mixture of MeOH (M) and water (W). A gradient sequence was executed as follows: The initial eluting condition was M–W (50:50, *v*/*v*), linearly changed to M–W (70:30, *v*/*v*) at 10 min, M–W (80:20, *v*/*v*) at 20 min, M–W (90:10, *v*/*v*) at 30 min, and M–W (100:0, *v*/*v*) at 40 min, and then kept the 100% MeOH condition over the next 20 min. The flow rate was set at 0.5 mL/min, the temperature of the column was maintained at 25 °C, and detection wavelengths were fixed at 220 nm. For the dry EtOAc extract from diverse sources, 0.25 mg was dissolved in 10 μL of methanol and filtered through a 0.45 m membrane filter prior to loading into the HPLC column, then the sample injection was performed manually with 10 μL injection volume.

### 3.4. Extraction and Isolation

The aquaculture soft coral of *L. crassum* (2015CSC-2) (622 g, wet weight) was freeze-dried, then the resulting dry material (213 g) was extracted exhaustively with EtOAc. The EtOAc extract was evaporated under reduced pressure to afford a residue (11.1 g). The residue was subjected to silica gel column chromatography, using mixtures of *n*-hexane, EtOAc, and acetone, with increasing polarity (*n*-hexane:EtOAc:acetone, 1:0:0, 100:1:0, 50:1:0, 20:1:0, 10:1:0, 5:1:0, 3:1:0, 2:1:0, 1:1:0, 1:2:0, 0:1:0, and 0:0:1) to yield 15 fractions. Fr-5 was fractioned with NP-HPLC eluting with *n*-hexane:EtOAc 10:1 to afford 12 subfractions (Fr-5-1−Fr-5-12). Subfraction Fr-5-10 was purified by normal-phase HPLC (*n*-hexane:dichloromethane:acetone 10:1:1) to afford **5** (42.3 mg). Subfractions Fr-5-12 was purified by normal-phase HPLC (*n*-hexane:EtOAc 15:1) to afford **2** (3.9 mg). Fr-6 was fractioned with NP-HPLC (*n*-hexane:dichloromethane:acetone 10:1:1), and then the following subfraction 3 was further separated using normal-phase HPLC (*n*-hexane:EtOAc 10:1) to yield **6** (35.7 mg) and **7** (25.9 mg). Fr-7 which showed a pure pattern in the NMR spectrum was simply purified with normal-phase HPLC (*n*-hexane:acetone 5:1) yielding 1264.5 mg of **8**. Fr-8 was subjected to silica gel column chromatography (dichloromethane:acetone 6:1) then the later subfraction 1 was purified with normal-phase (*n*-hexane:EtOAc 4:1) and reversed-phase (70% MeOH) HPLC to afford **9** (6.4 mg), **12** (5.8 mg)**,** and **13** (5.1 mg). Another subfraction 3 was subjected to normal-phase HPLC (*n*-hexane:acetone 4:1) to obtain **4** (2.9 mg), **10** (34.3 mg), and **11** (7.9 mg). Fr-10 was purified by normal-phase HPLC (*n*-hexane:EtOAc 7:1) to yield **1** (5.9 mg) and **3** (34.0 mg).

Crassumtocopherol C (**1**): yellow oil; ${\left[\alpha \right]}_{\;D}^{24}$ = −68 (*c* 0.17, CHCl_3_); IR (neat, CHCl_3_) v_max_ 3384, 2937, 2870, 1457, 1419, 1377, 1258, 1216 cm^−1^; ^13^C (CDCl_3_, 125 MHz) and ^1^H (CDCl_3_, 500 MHz) NMR data, see Table 1; ESIMS *m*/*z* 485 [M + Na]^+^; HRESIMS *m*/*z* 485.3599 ± 0.0005 [M + Na]^+^ (calcd for C_29_H_50_O_4_Na, 485.36013).

Culobophylin D (**2**): white, amorphous powder; ${\left[\alpha \right]}_{\;D}^{24}$ = +47 (*c* 0.20, CHCl_3_); IR (neat, CHCl_3_) *v*_max_ 3299, 3050, 1642, 1433, 1379, 1350, 1218 cm^−1^; ^13^C (CDCl_3_, 125 MHz) and ^1^H (CDCl_3_, 500 MHz) NMR data, see Table 2; ESIMS *m*/*z* 327 [M + Na]^+^; HRESIMS *m*/*z* 327.2296 ± 0.0005 [M + Na]^+^ (calcd for C_20_H_32_O_2_Na, 327.22945).

Culobophylin E (**3**): colorless oil; ${\left[\alpha \right]}_{\;D}^{28}$ = −227 (*c* 0.10, CHCl_3_); UV (MeOH) λ_max_ (log *ε*) 203 (3.13) nm; IR (neat, CHCl_3_) v_max_ 3417, 2924, 1693, 1621, 1454, 1377, 1240, 1215 cm^−1^; ^13^C (CDCl_3_, 125 MHz) and ^1^H (CDCl_3_, 500 MHz) NMR data, see Table 3; ESIMS *m*/*z* 341 [M + Na]^+^; HRESIMS *m*/*z* 341.2086 ± 0.0005 [M + Na]^+^ (calcd for C_20_H_30_O_3_Na, 341.20872).

### 3.5. Bioassay Materials

American Type Culture Collection (ATCC, Manassas, VA, USA) was the source for all of thecell lines. Cell lines were kept at 37 °C in a humidified atmosphere of 5% CO_2_ in RPMI 1640 medium supplemented with 10% fetal calf serum, 2 mM glutamine, and antibiotics (100 units/mL of penicillin and 100 μg/mL of streptomycin). Trypan blue, fetal calf serum (FCS), RPMI 1640 medium, streptomycin, and penicillin G were purchased from GibcoBRL (Gaithersburg, MD, USA). 3-(4,5-Dimethylthiazol-2-yl)-2,5-diphenyl-tetrazolium bromide (MTT), dimethyl sulfoxide (DMSO) and all other chemicals were obtained from Sigma-Aldrich (St. Louis, MO, USA).

### 3.6. MTT Cell Proliferative Assay

Culture plates (96-well) were used in the MTT assay. Cells were seeded at 4 × 10^4^ per well and then treated with different concentrations of the tested compounds [19]. The cytotoxic effect of the tested compound was determined by MTT cell proliferation assay (thiazolyl blue tetrazolium bromide, Sigma-M2128) for 24, 48, or 72 h. ELISA reader (Anthoslabtec Instrument, Salzburg, Austria) was used to measure light absorbance values (OD = OD_570_ − OD_620_) at 570 and 620 nm. The concentration that caused 50% inhibition (IC_50_) was calculated. These results were expressed as a percentage of the control ± SD established from n = 4 wells per experiment from three independent experiments.

## 4. Conclusions

Tracing unique chromatographic peaks of the aquaculture *L. crassum* EtOAc extract led to the isolation of a series of cembrane-type diterpenoids. One novel tocopherol, crassumtocopherol C (**1**) and two new cembranoids, culobophylin D (**2**) and E (**3**) were identified along with ten previously reported cembranoids. Compound **2** was recognized as the first cembranoid exhibiting saturated internal C_4_-O-C_14_ linkage six-membered ring. All of the isolates (except **3**) were evaluated for their anti-proliferative effect against leukemia cells. The major two cembranoids, compounds **8** and **10**, exhibited the most potent activity and were deduced to be the main contributors to the anti-proliferative property of the aquaculture *L*. *crassum* EtOAc extract. These results suggested the importance of developing efficient aquaculture protocols to provide a continuous and sustainable supply of biologically active secondary metabolites. 

## Figures and Tables

**Table 1:** ^1^H, ^13^C, ^1^H–^1^H COSY, and HMBC NMR data of **1**.

Position	*δ*_H_ (*J* in Hz) *^a^*	*δ*_C_ (Mult.) *^b^*	^1^H–^1^H COSY	HMBC
2		74.4 (C)		
3	1.79 m	31.7 (CH_2_)	H-4	C-4a, C-2
4	2.61 t (7.0)	20.7 (CH_2_)	H-3	C-4a, C-8a, C-3, C-4
4a		117.4 (C)		
5		118.7 (C)		
6		145.5 (C)		
7		122.6 (C)		
8		121.2 (C)		
8a		144.5 (C)		
1’	1.47 m; 1.56 m	39.2 (CH_2_)	H-2’	C-2, C-2’
2’	1.40 m; 1.47 m	20.8 (CH_2_)	H-1’, H-3’	
3’	1.09 m; 1.26 m	37.3 (CH_2_)	H-2’, H-4’	C-2’
4’	1.42 m	32.4 (CH)	H-3’, H-5’, H-4’-Me	
5’	1.14 m; 1.26 m	37.3 (CH_2_)	H-4’, H-6’	C-6’
6’	1.26 m	21.3 (CH_2_)	H-5’	
7’	1.52 m; 1.65 m	42.8 (CH_2_)		C-6’, C-8’
8’		72.6 (C)		
9’	1.40 m	38.2 (CH_2_)	H-10’	C-7’, C-8’, C-11’
10’	1.48 m; 1.56 m	28.2 (CH_2_)	H-9’, H-11’	C-9’, C-11’
11’	3.36 m	77.4 (CH)	H-10’	
12’	1.69 m	33.7 (CH)	H-11’, H-13’, H-12’-Me	
13’	0.93 d (7.0)	19.8 (CH_3_)	H-12’	C-11’, C-12’
2-Me	1.24 s	24.0 (CH_3_)		C-2, C-3, C-1’
5-Me	2.11 s	11.8 (CH_3_)		C-4a, C-5, C-6
7-Me	2.17 s	12.2 (CH_3_)		C-6, C-7, C-8
8-Me	2.11 s	11.3 (CH_3_)		C-7, C-8, C-8a
4’-Me	0.86 d (6.5)	19.6 (CH_3_)		C-3’, C-4’, C-5’
8’-Me	1.17 s	26.7 (CH_3_)		C-7’, C-8’, C-9’
12’-Me	0.93 d (7.0)	17.3 (CH_3_)	H-12’	C-11’, C-12’

Spectra recorded at *^a^* 500 and *^b^* 125 MHz in CDCl_3_.

**Table 2:** ^1^H, ^13^C, ^1^H–^1^H COSY, and HMBC NMR data of **2**.

Position	*δ*_H_ (*J* in Hz) *^a^*	*δ*_C_ (Mult.) *^b^*	^1^H–^1^H COSY	HMBC
1	2.19 m	46.1 (CH)	H-2, H-14	
2	1.86 m	33.6 (CH_2_)	H-3	C-1, C-4, C-14
3	3.72 dd (11.0, 6.0)	71.2 (CH)	H-2	C-2, C-4, C-5, C-18
4		76.9 (C)		C-3, C-4, C-6
5	1.48 m	39.4 (CH_2_)	H-6	
6	1.97 m; 2.32 m	23.2 (CH_2_)	H-5, H-7	
7	5.01 br d (11.0)	129.7 (CH)	H-6	C-6
8		128.4 (C)		
9	1.95 m; 2.18 m	39.8 (CH_2_)	H-10	C-7, C-8, C-10
10	1.92 m; 2.33 m	25.8 (CH_2_)	H-9, H-11	C-11
11	4.87 brs	128.0 (CH)	H-10	C-10
12		130.8 (C)		
13	1.78 m; 2.21 m	43.6 (CH_2_)	H-14	C-1, C-12, C-14
14	3.61 dt (11.5, 3.0)	66.5 (CH)	H-13	
15		145.3 (C)		
16	1.81 s	24.4 (CH_3_)	H-17	C-1, C-15, C-17
17	4.88 br s; 4.97 d (1.5)	113.7 (CH_2_)	H-16	C-1, C-15, C-16
18	1.08 s	12.7 (CH_3_)		C-3, C-4, C-5
19	1.56 s	15.4 (CH_3_)	H-9	C-7, C-8, C-9
20	1.50 s	15.2 (CH_3_)		C-11, C-12, C-13

Spectra recorded at *^a^* 500 and *^b^* 125 MHz in CDCl_3_.

**Table 3:** ^1^H, ^13^C, ^1^H–^1^H COSY, and HMBC NMR data of **3**.

Position	*δ*_H_ (*J* in Hz) *^a^*	*δ*_C_ (Mult.) *^b^*	^1^H–^1^H COSY	HMBC
1	2.77 m	34.2 (CH)	H-2, H-14	C-2, C-3, C-13, C-14, C-15, C-16, C-17
2	1.51 m; 1.87 m	34.3 (CH_2_)	H-3	C-3, C-4, C-5, C-14, C-15, C-16
3	2.84 dd (9.5, 3.0)	62.9 (CH)	H-2	C-5
4		60.9 (C)		C-3, C-4, C-6
5	1.29 m	38.3 (CH_2_)	H-6	C-3, C-4
6	2.19 m	24.5 (CH_2_)	H-5, H-7	C-5, C-7, C-8
7	5.11 t (6.5)	124.8 (CH)	H-6	C-5, C-6, C-19
8		133.2 (C)		
9	2.18 m	39.5 (CH_2_)	H-10	C-7, C-8, C-10, C-11, C-12
10	2.19 m	23.7 (CH_2_)	H-9, H-11	C-8, C-9, C-11, C-12
11	5.16 t (6.5)	123.8 (CH)	H-10	C-9, C-10, C-20
12		135.2 (C)		
13	2.10 m	35.0 (CH_2_)	H-14	C-1, C-11, C-12, C-14
14	1.77 m	30.7 (CH_2_)	H-13	C-1, C-2, C-13, C-15
15		144.1 (C)		
16	5.65 s, 6.36 d (1.0)	126.3 (CH_2_)		C-1, C-15, C-17
17		170.2 (C)		
18	1.23 s	16.9 (CH_3_)		C-3, C-4, C-5
19	1.58 s	16.9 (CH_3_)		C-7, C-8, C-9
20	1.62 s	15.6 (CH_3_)		C-11, C-12, C-13

Spectra recorded at *^a^* 500 and *^b^* 125 MHz in CDCl_3_.

**Table 4:** Anti-proliferative effect of the isolates from the cultured soft coral of *L. crassum*.

Compounds	IC_50_ (μM) of Leukemia Cell Lines at 72 h			
	K562	Molt 4	U937	Sup-T1
EtOAc extract	3.3	1.0	1.7	2.2
1	34.0	NA *^a^*	NA *^a^*	23.3
2	NA *^a^*	NA *^a^*	NA *^a^*	NA *^a^*
4	NA *^a^*	NA *^a^*	NA *^a^*	35.8
5	16.3	12.3	NA *^a^*	4.6
6	11.3	6.2	15.8	5.2
7	18.1	8.4	4.4	8.3
8	3.3	1.2	7.1	1.5
9	15.3	11.6	32.0	10.2
10	4.5	2.9	7.0	4.5
11	3.3	2.3	5.2	6.2
12	12.3	4.8	10.9	6.1
13	13.0	7.0	23.3	6.6
Doxorubicin *^b^*	0.13	0.02	0.04	0.09

*^a^* NA (nonactive): IC_50_ > 60 μM for 72 h; *^b^* Positive control.

## Supplementary Materials

Supplementary materials according to this paper are available online at www.mdpi.com/1660-3397/16/1/15/s1.
