# Supplementary material for: Aquaculture Soft Coral Lobophytum crassum as a Producer of Anti-Proliferative Cembranoids
Source: Mar Drugs. 2018 Jan 7;16(1):15. doi: 10.3390/md16010015 (PMC5793063; doi:10.3390/md16010015)
Supplement: Supplementary file 1 [file marinedrugs-16-00015-s001.pdf]

Aquaculture Soft Coral *Lobophytum crassum* as a Producer of Anti-Proliferative Cembranoids

Bo-Rong Peng [1,2], Mei-Chin Lu [3,4], Mohamed El-Shazly [5,6], Shwu-Li Wu [7], Kuei-Hung Lai [3,*], Jui-Hsin Su [3,4,*]

[1] Doctoral Degree Program in Marine Biotechnology, National Sun Yat-Sen University (NSYSU), 70 Lien-Hai Road, Kaohsiung 80424, Taiwan; pengpojung@gmail.com

[2] Doctoral Degree Program in Marine Biotechnology, Academia Sinica, 128 Academia Road, Section 2, Nankang, Taipei 11529, Taiwan; pengpojung@gmail.com

[3] National Museum of Marine Biology & Aquarium, Pingtung 94450, Taiwan; jinx6609@nmmba.gov.tw (M.-C.L.); mos19880822@gmail.com (K.-H.L.); x2219@nmmba.gov.tw (J.-H.S.)

[4] Graduate Institute of Marine Biology, National Dong Hwa University, Pingtung 94450, Taiwan; jinx6609@nmmba.gov.tw (M.-C.L.); x2219@nmmba.gov.tw (J.-H.S.)

[5] Department of Pharmacognosy and Natural Products Chemistry, Faculty of Pharmacy, Ain-Shams University, Organization of African Unity Street, Abassia, Cairo 115, Egypt; mohamed.elshazly@pharma.asu.edu.eg

[6] Department of Pharmaceutical Biology, Faculty of Pharmacy and Biotechnology, German University in Cairo, Cairo 11432, Egypt; elshazly444@googlemail.com

[7] General Study Center, National Kaohsiung Marine University, Kaohsiung 80543, Taiwan; wusl@webmail.nkmu.edu.tw

* Authors to whom correspondence should be addressed;
E-Mails: mos19880822@gmail.com (K.-H.L.); x2219@nmmba.gov.tw (J.-H.S.)
Tel.: +886-8-882-5001#1326 (K.-H.L. and J.-H.S.)

Supporting information

Table of Contents

Figure S1: ^{1}H NMR (500 MHz, $CDCl_3$) spectrum of **1**. ---------- 3
Figure S2: ^{13}C NMR (125 MHz, $CDCl_3$) spectrum of **1**. ---------- 4
Figure S3: DEPT NMR (125 MHz, $CDCl_3$) spectrum of **1**. ---------- 5
Figure S4: COSY NMR (500 MHz, $CDCl_3$) spectrum of **1**. ---------- 6
Figure S5: NOESY NMR (500 MHz, $CDCl_3$) spectrum of **1**. ---------- 7
Figure S6: HSQC NMR (500 MHz, $CDCl_3$) spectrum of **1**. ---------- 8
Figure S7: HMBC NMR (500 MHz, $CDCl_3$) spectrum of **1**. ---------- 9
Figure S8: HRESIMS spectrum of **1**. ---------- 10
Figure S9: IR spectrum of **1**. ---------- 11
Figure S10: ^{1}H NMR (500 MHz, $CDCl_3$) spectrum of **2**. ---------- 12
Figure S11: ^{13}C NMR (125 MHz, $CDCl_3$) spectrum of **2**. ---------- 13
Figure S12: DEPT NMR (125 MHz, $CDCl_3$) spectrum of **2**. ---------- 14
Figure S13: COSY NMR (500 MHz, $CDCl_3$) spectrum of **2**. ---------- 15
Figure S14: NOESY NMR (500 MHz, $CDCl_3$) spectrum of **2**. ---------- 16
Figure S15: HSQC NMR (500 MHz, $CDCl_3$) spectrum of **2**. ---------- 17
Figure S16: HMBC NMR (500 MHz, $CDCl_3$) spectrum of **2**. ---------- 18
Figure S17: HRESIMS spectrum of **2**. ---------- 19
Figure S18: IR spectrum of **2**. ---------- 20
Figure S19: ^{1}H NMR (500 MHz, $CDCl_3$) spectrum of **3**. ---------- 21
Figure S20: ^{13}C NMR (125 MHz, $CDCl_3$) spectrum of **3**. ---------- 22
Figure S21: DEPT NMR (125 MHz, $CDCl_3$) spectrum of **3**. ---------- 23
Figure S22: COSY NMR (500 MHz, $CDCl_3$) spectrum of **3**. ---------- 24
Figure S23: NOESY NMR (500 MHz, $CDCl_3$) spectrum of **3**. ---------- 25
Figure S24: HSQC NMR (500 MHz, $CDCl_3$) spectrum of **3**. ---------- 26
Figure S25: HMBC NMR (500 MHz, $CDCl_3$) spectrum of **3**. ---------- 27
Figure S26: HRESIMS spectrum of **3**. ---------- 28
Figure S27: IR spectrum of **3**. ---------- 29

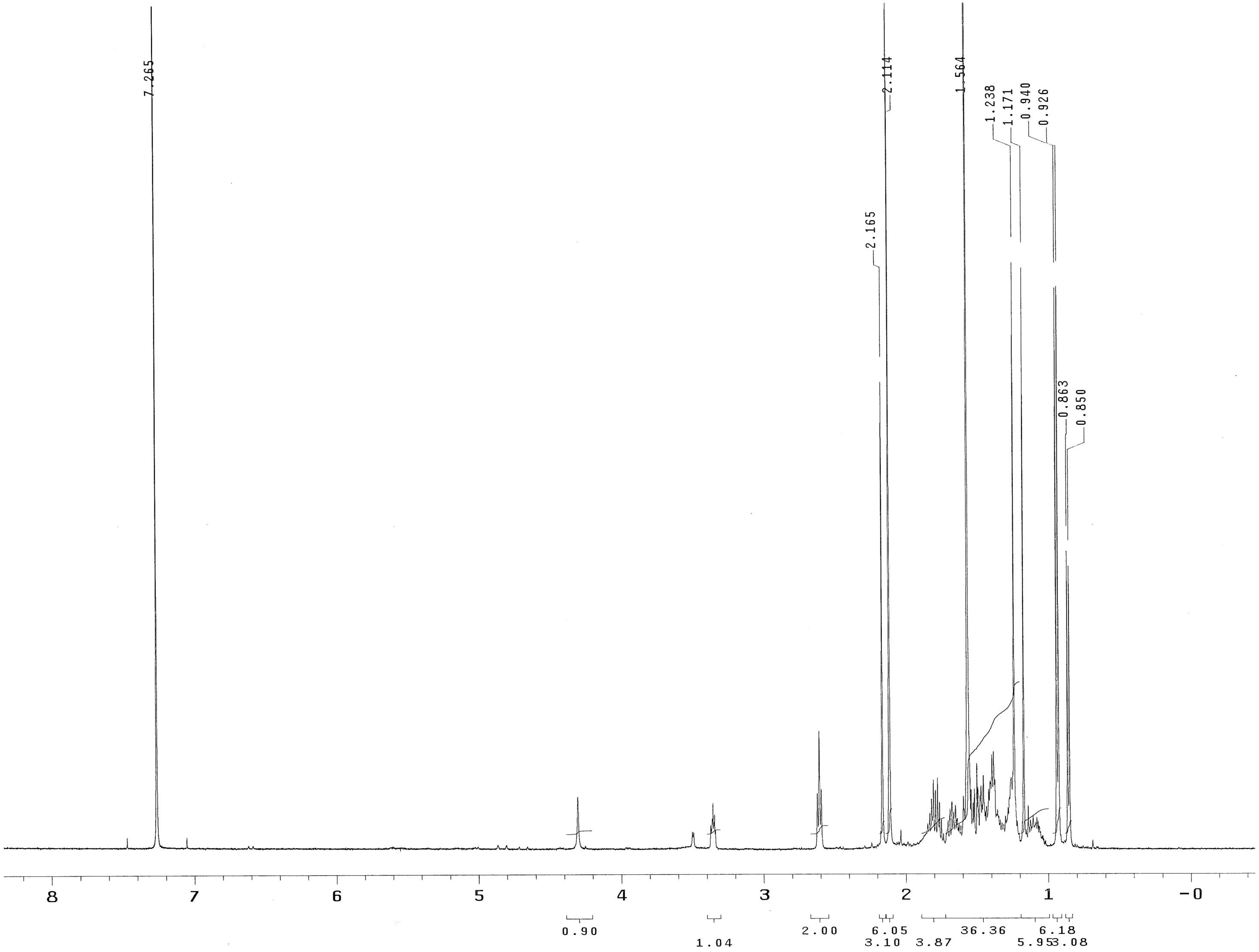

Figure S1: ^{1}H NMR (500 MHz, $CDCl_3$) spectrum of **1**.

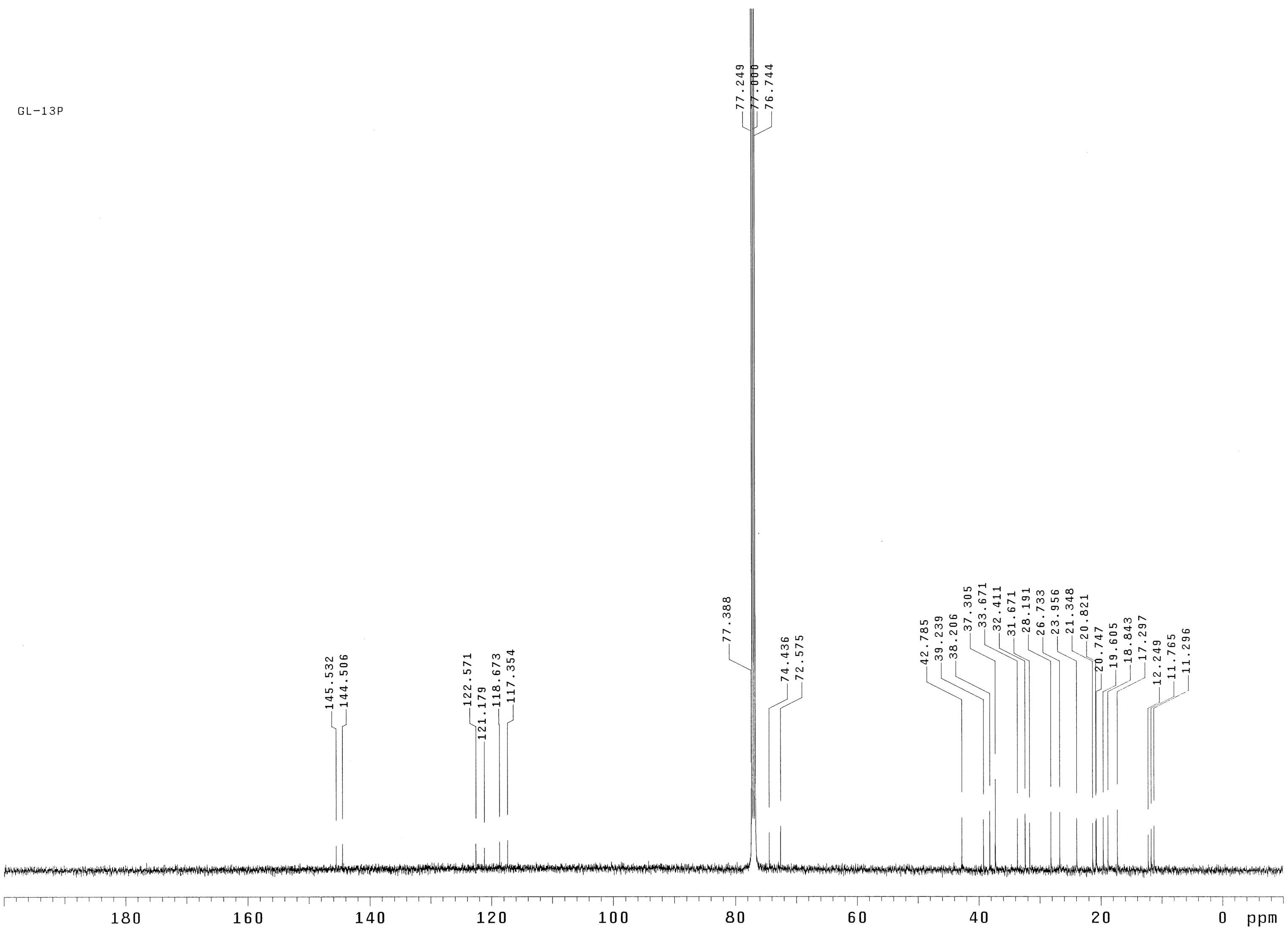

Figure S2: ^{13}C NMR (125 MHz, $CDCl_3$) spectrum of **1**.

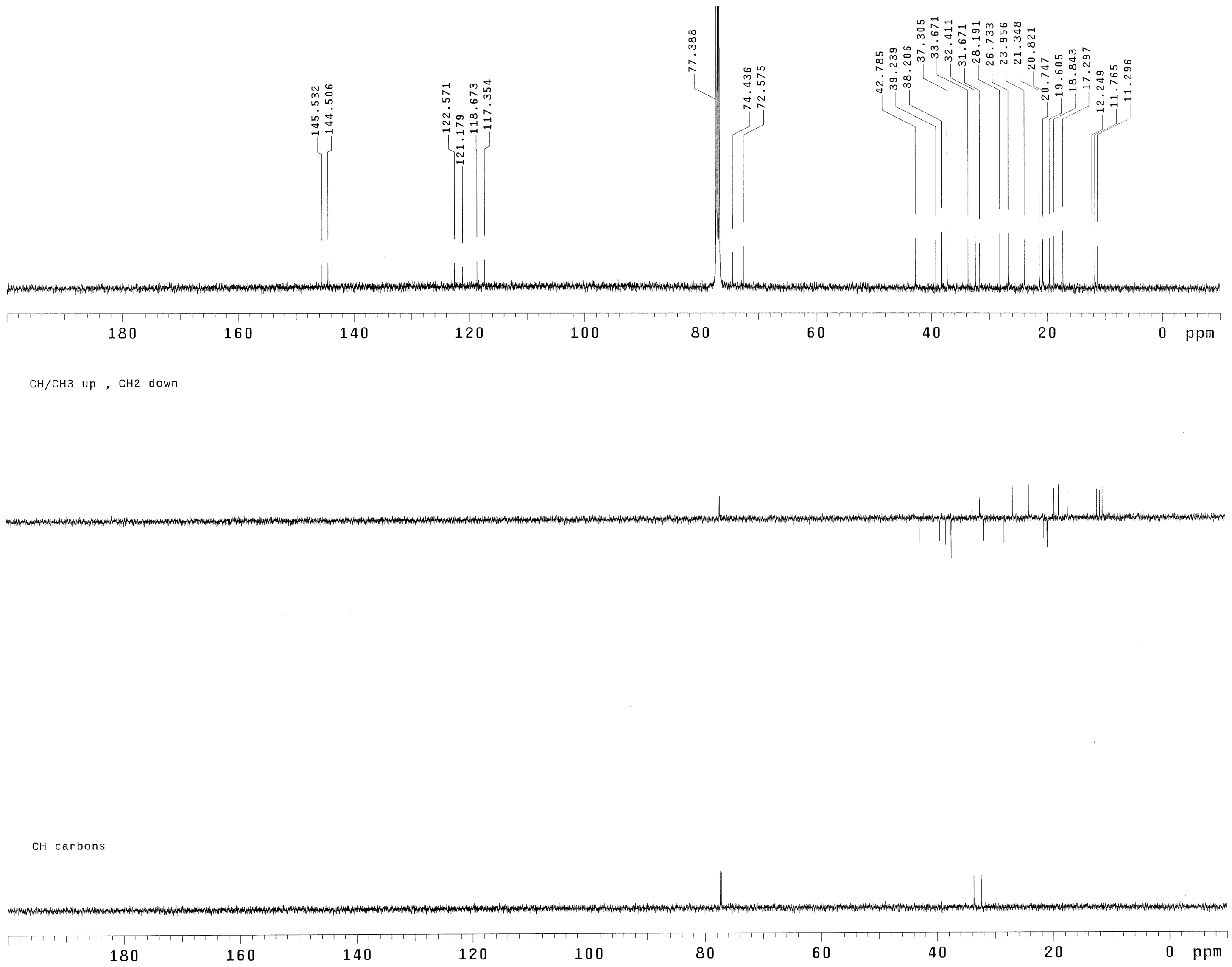

Figure S3: DEPT NMR (125 MHz, $CDCl_3$) spectrum of **1**.

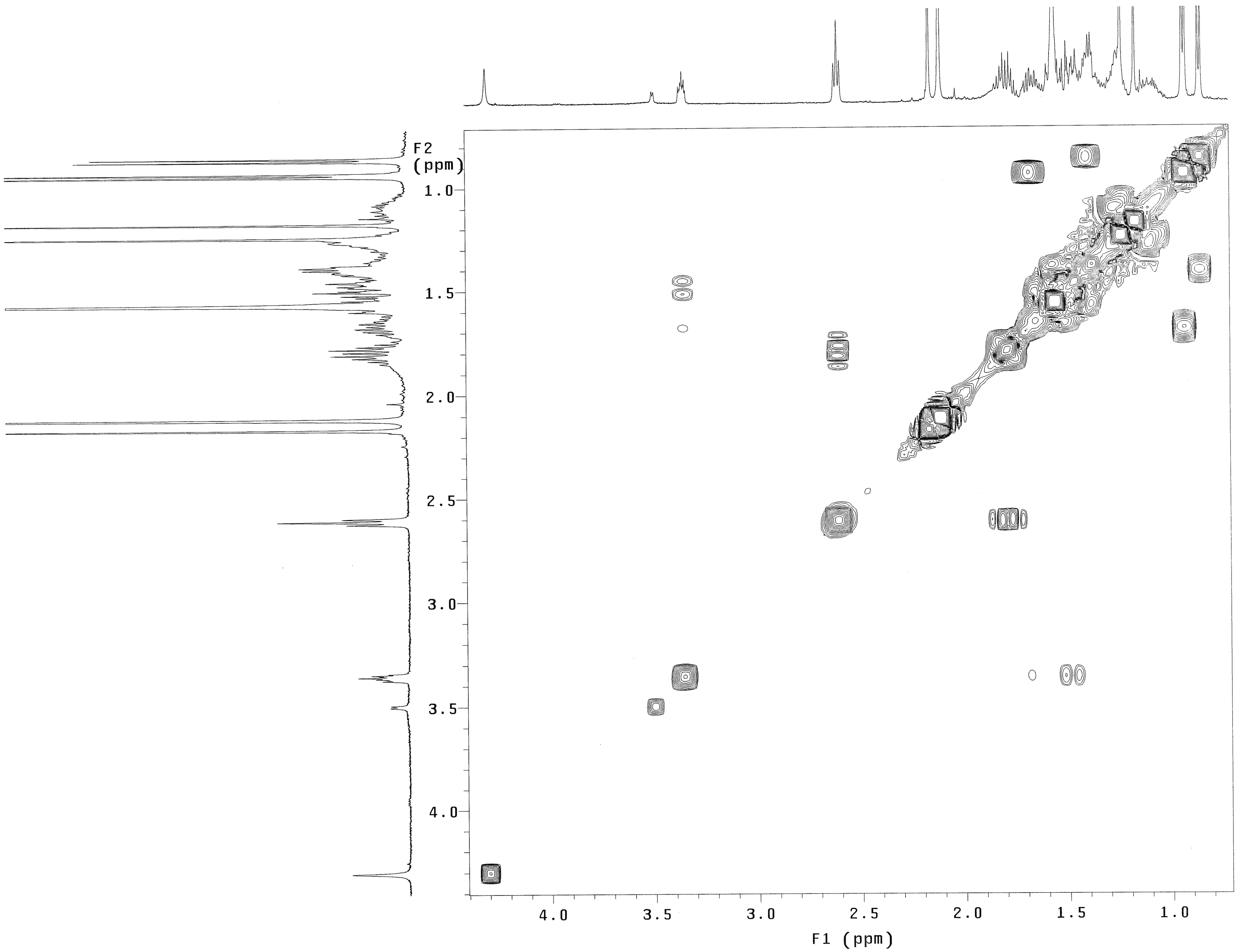

Figure S4: COSY NMR (500 MHz, $CDCl_3$) spectrum of **1**.

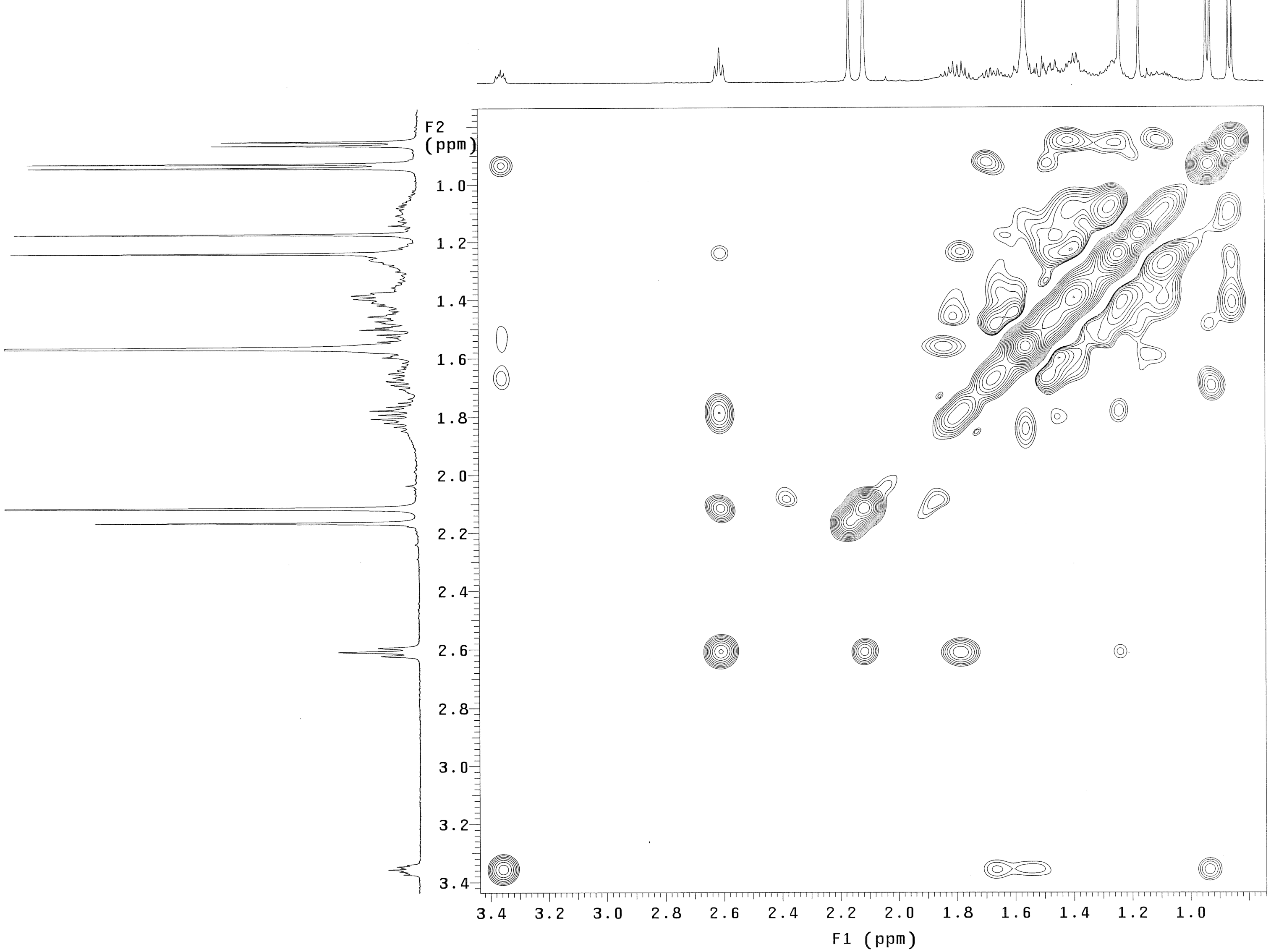

Figure S5: NOESY NMR (500 MHz, $CDCl_3$) spectrum of **1**.

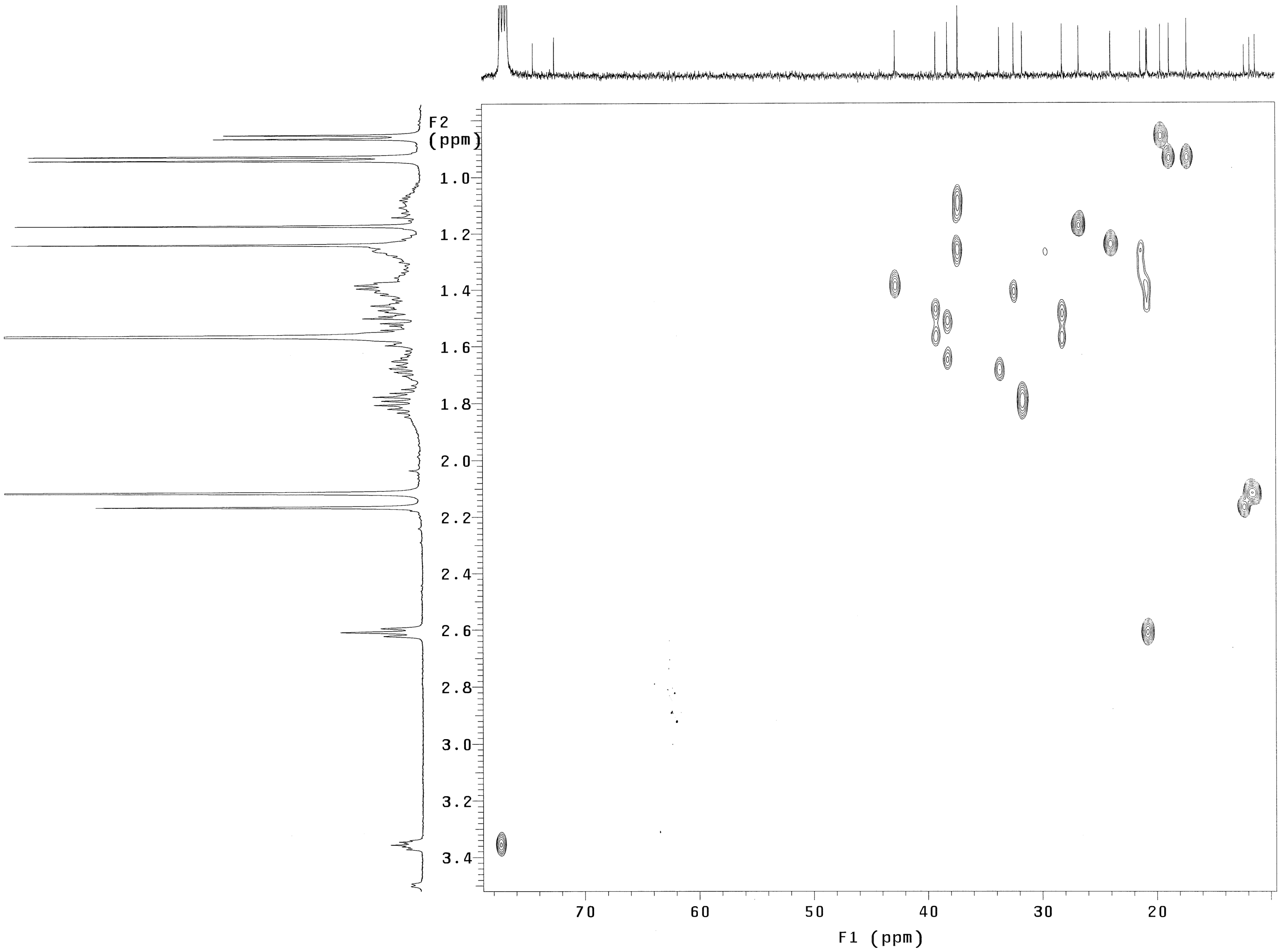

Figure S6: HSQC NMR (500 MHz, $CDCl_3$) spectrum of **1**.

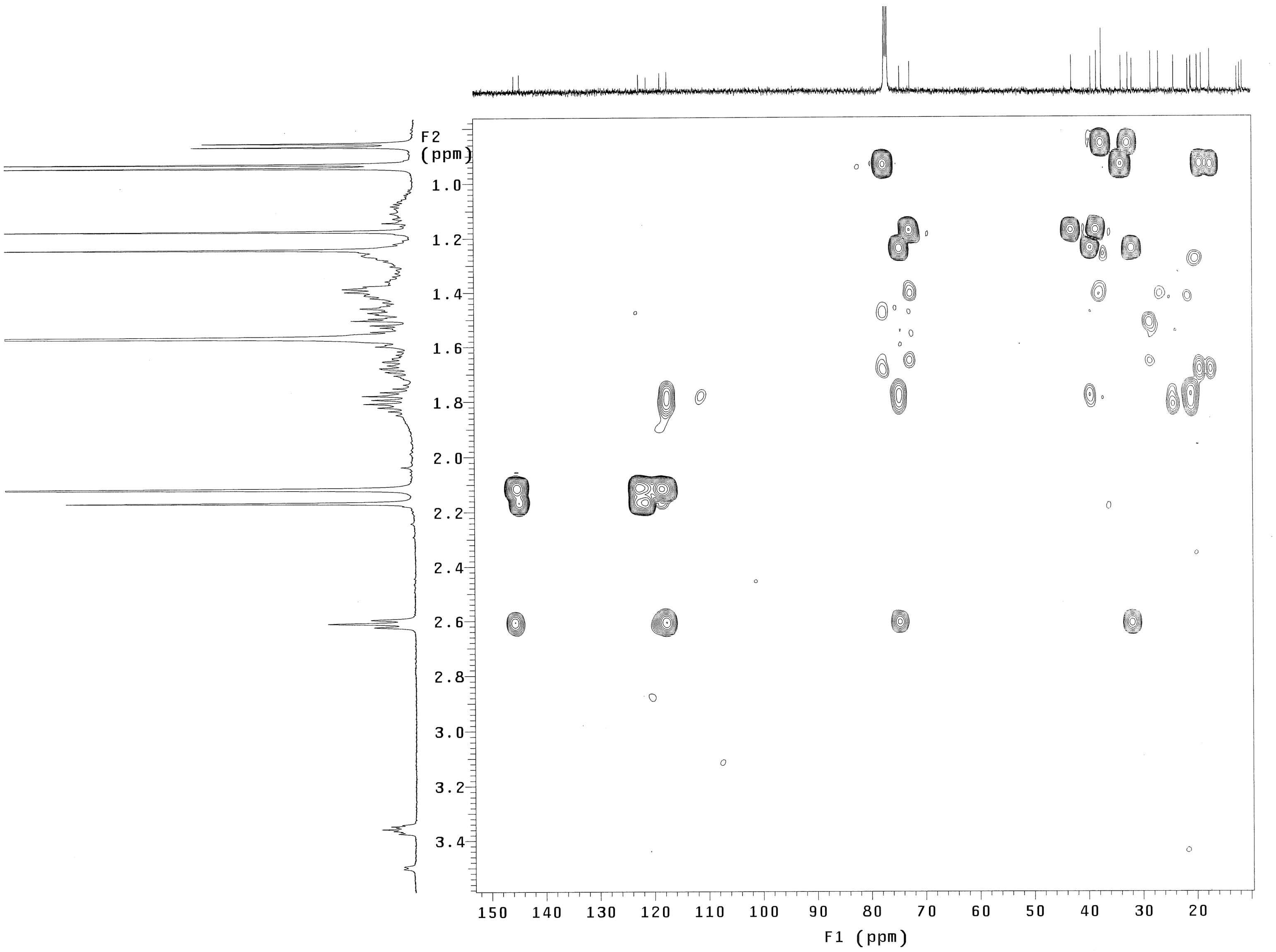

Figure S7: HMBC NMR (500 MHz, $CDCl_3$) spectrum of **1**.

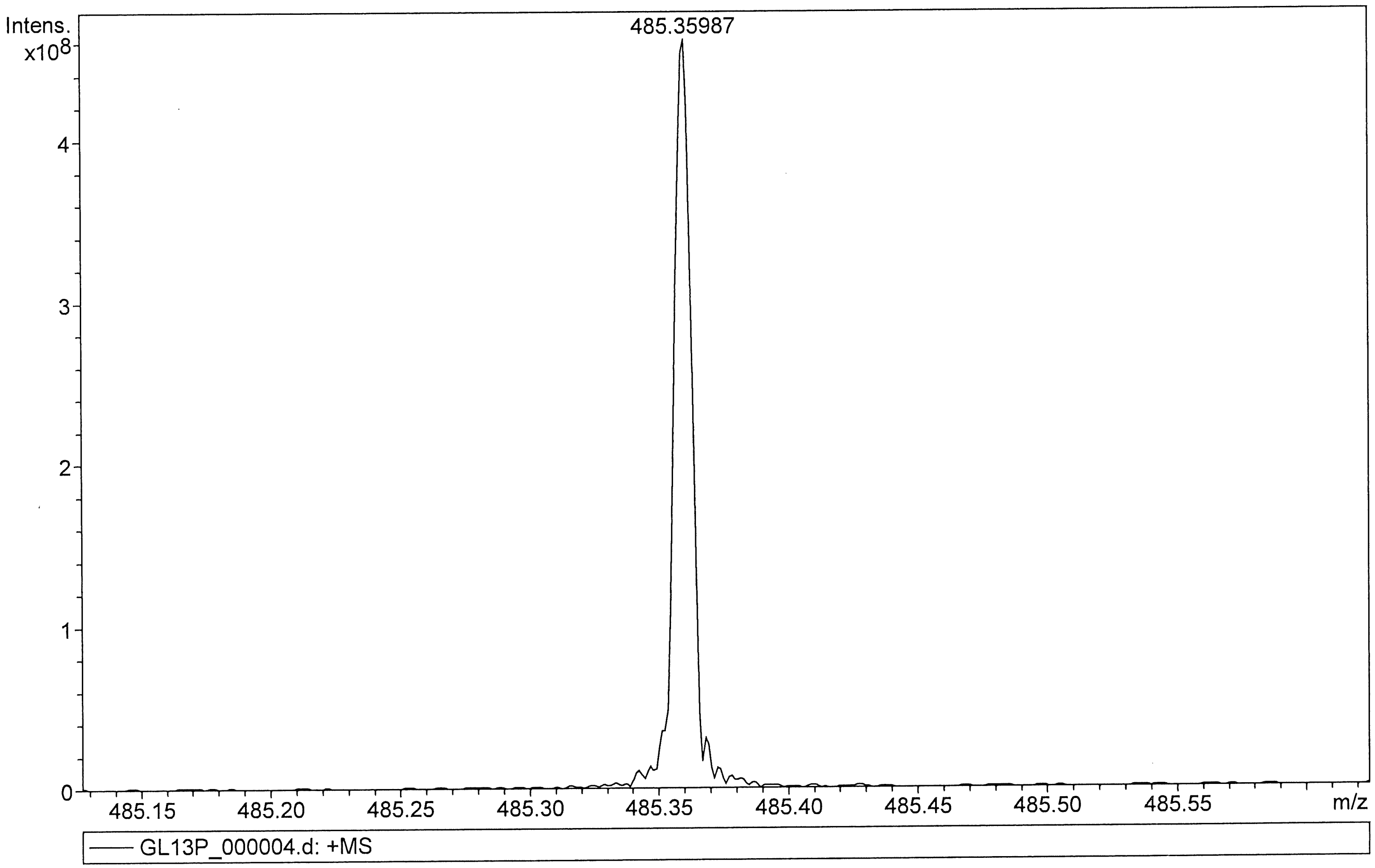

Meas. m/z	#	Formula	Score	m/z	err [mDa]	err [ppm]	mSigma	rdb	e⁻ Conf	N-Rule
485.35987	1	C 29 H 50 Na O 4	100.00	485.36013	0.26	0.54	3.9	4.5	even	ok

Figure S8: HRESIMS spectrum of **1**.

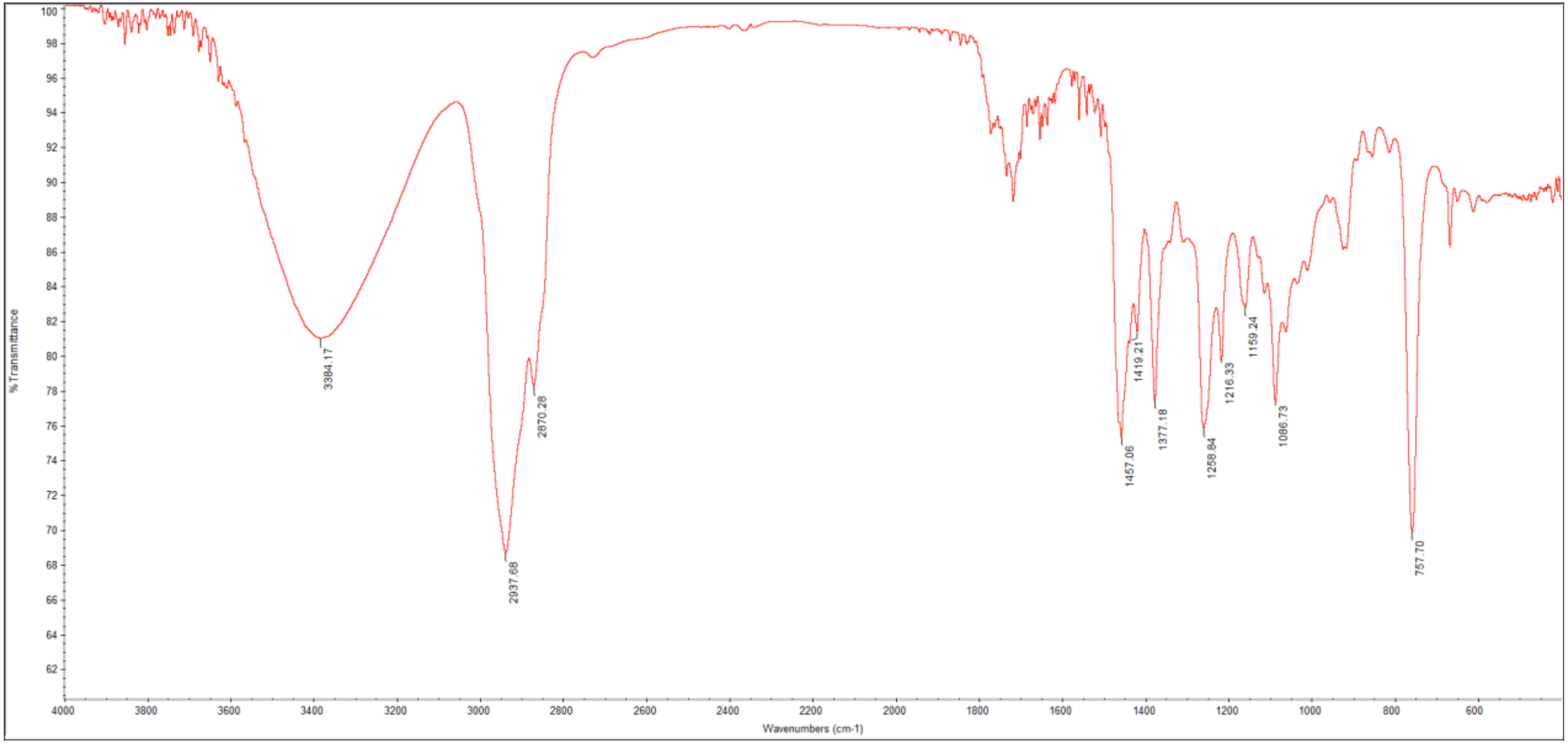

Figure S9: IR spectrum of **1**.

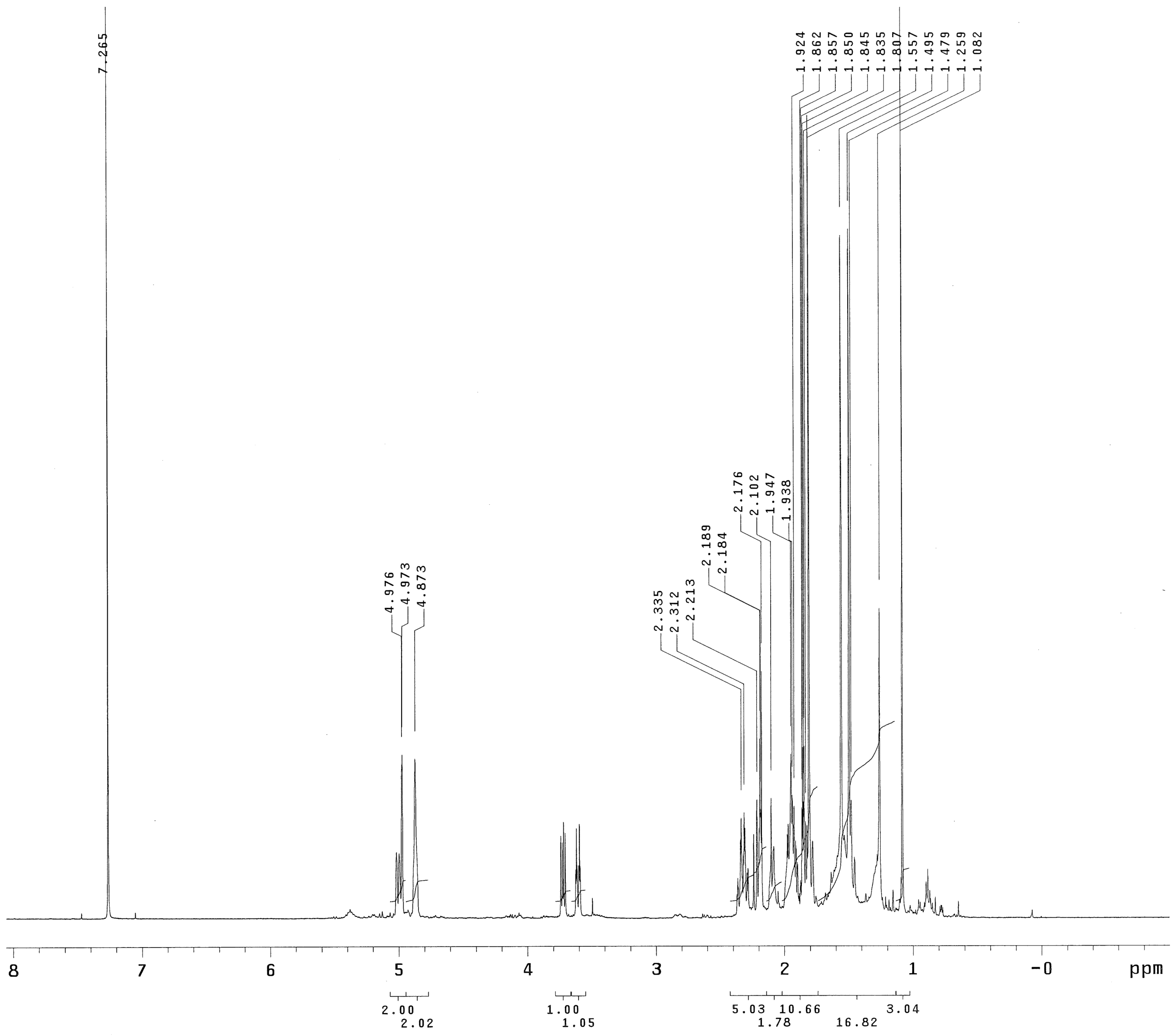

Figure S10: ^{1}H NMR (500 MHz, $CDCl_3$) spectrum of **2**.

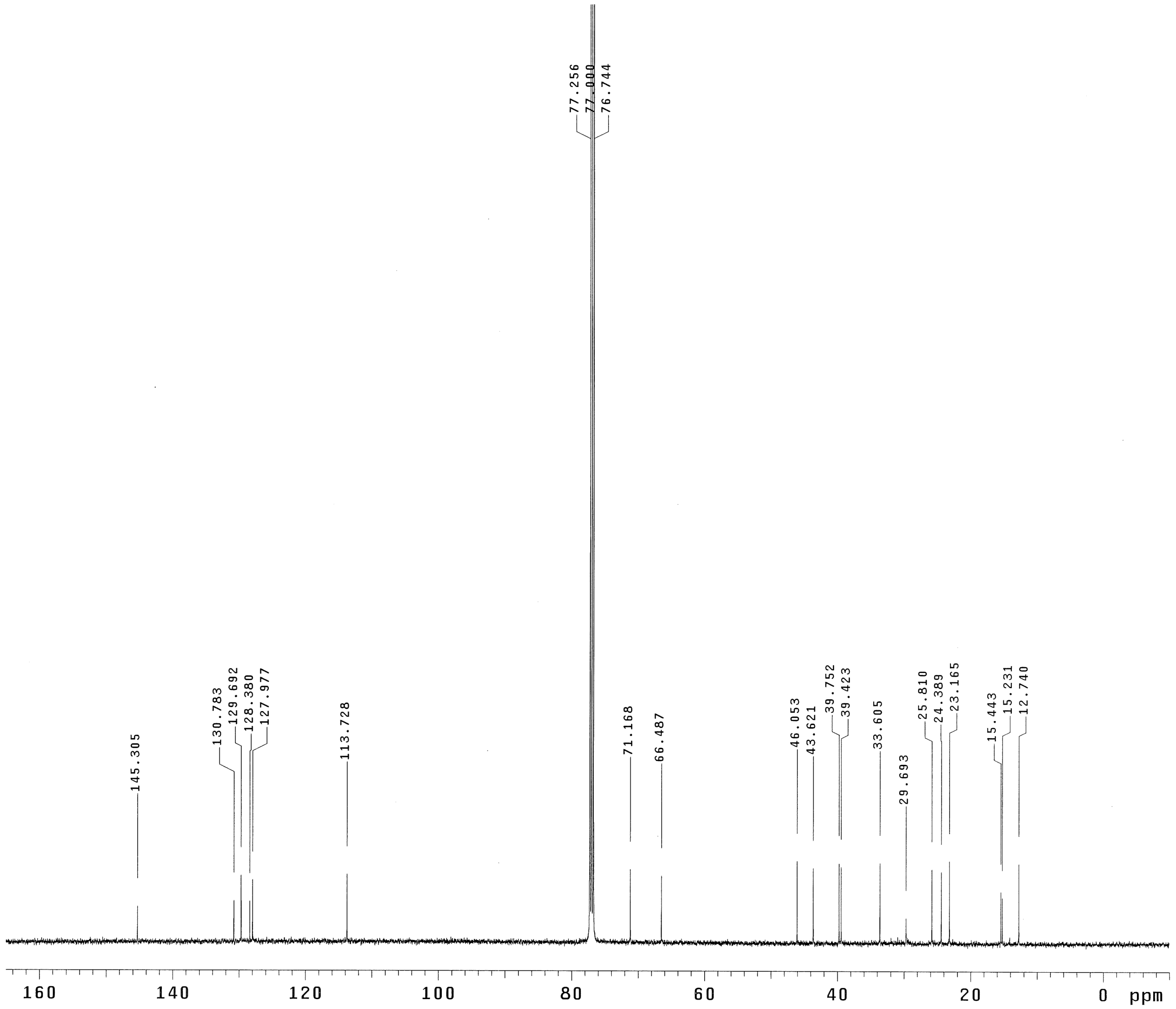

Figure S11: ^{13}C NMR (125 MHz, $CDCl_3$) spectrum of **2**.

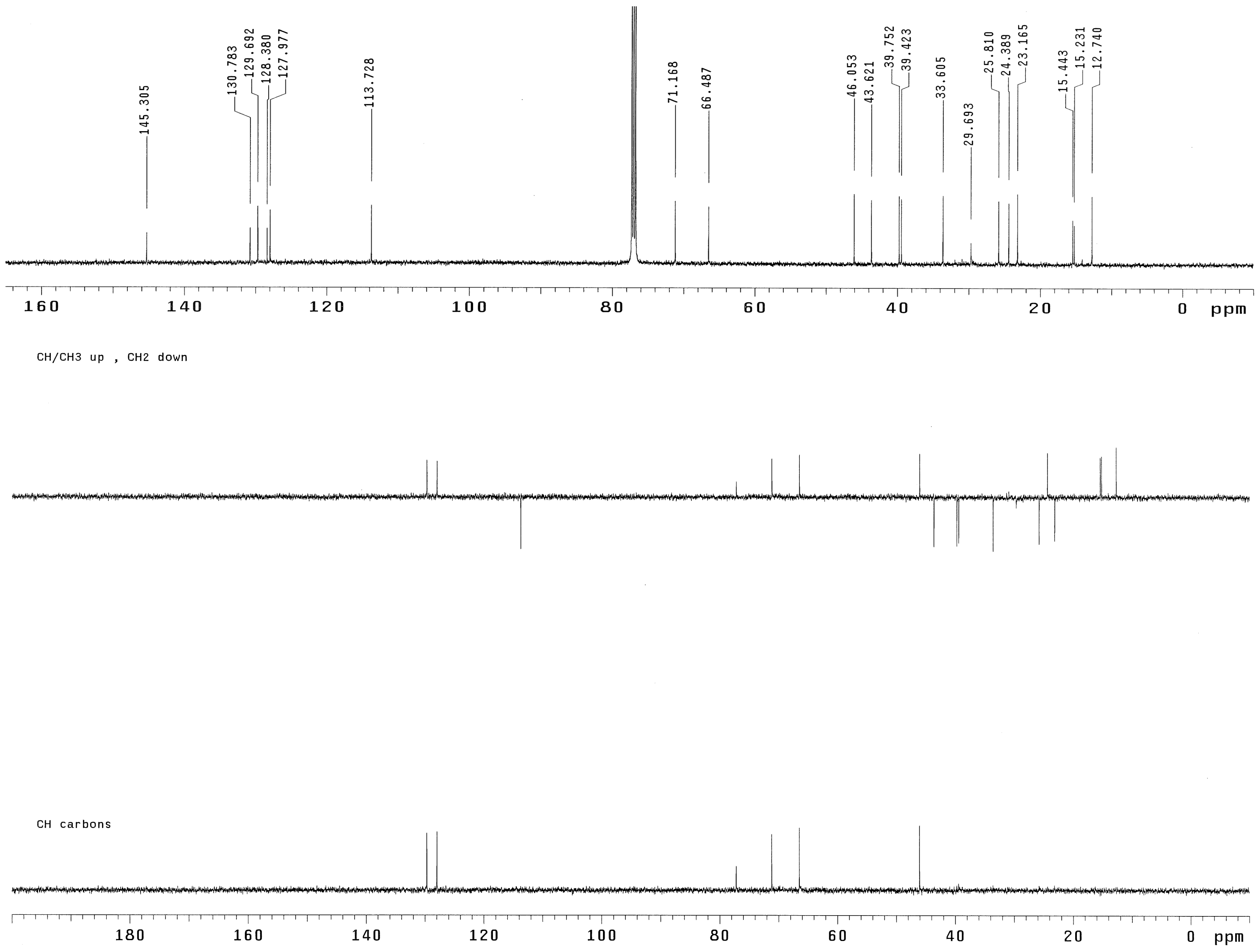

Figure S12: DEPT NMR (125 MHz, $CDCl_3$) spectrum of **2**.

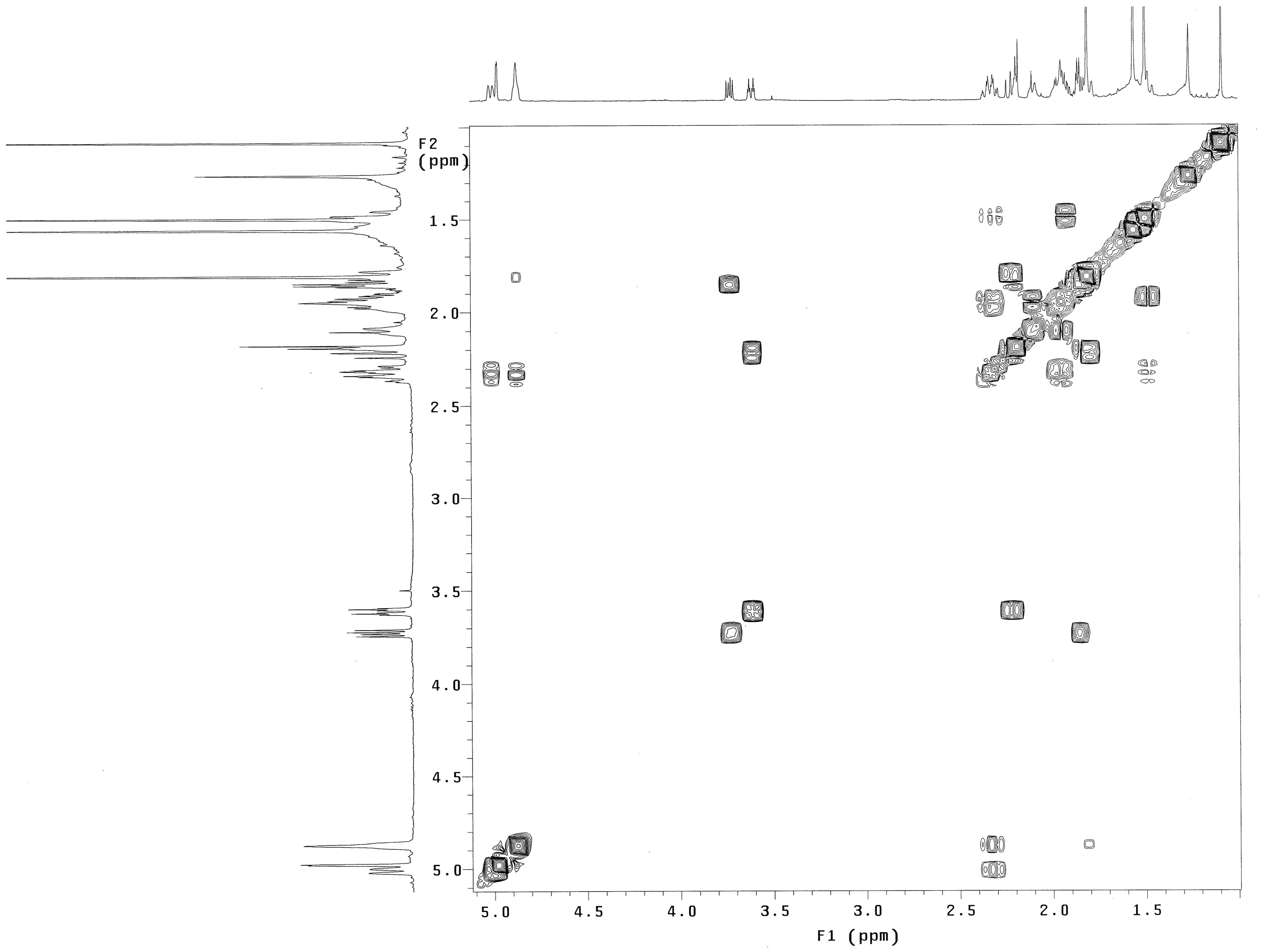

Figure S13: COSY NMR (500 MHz, $CDCl_3$) spectrum of **2**.

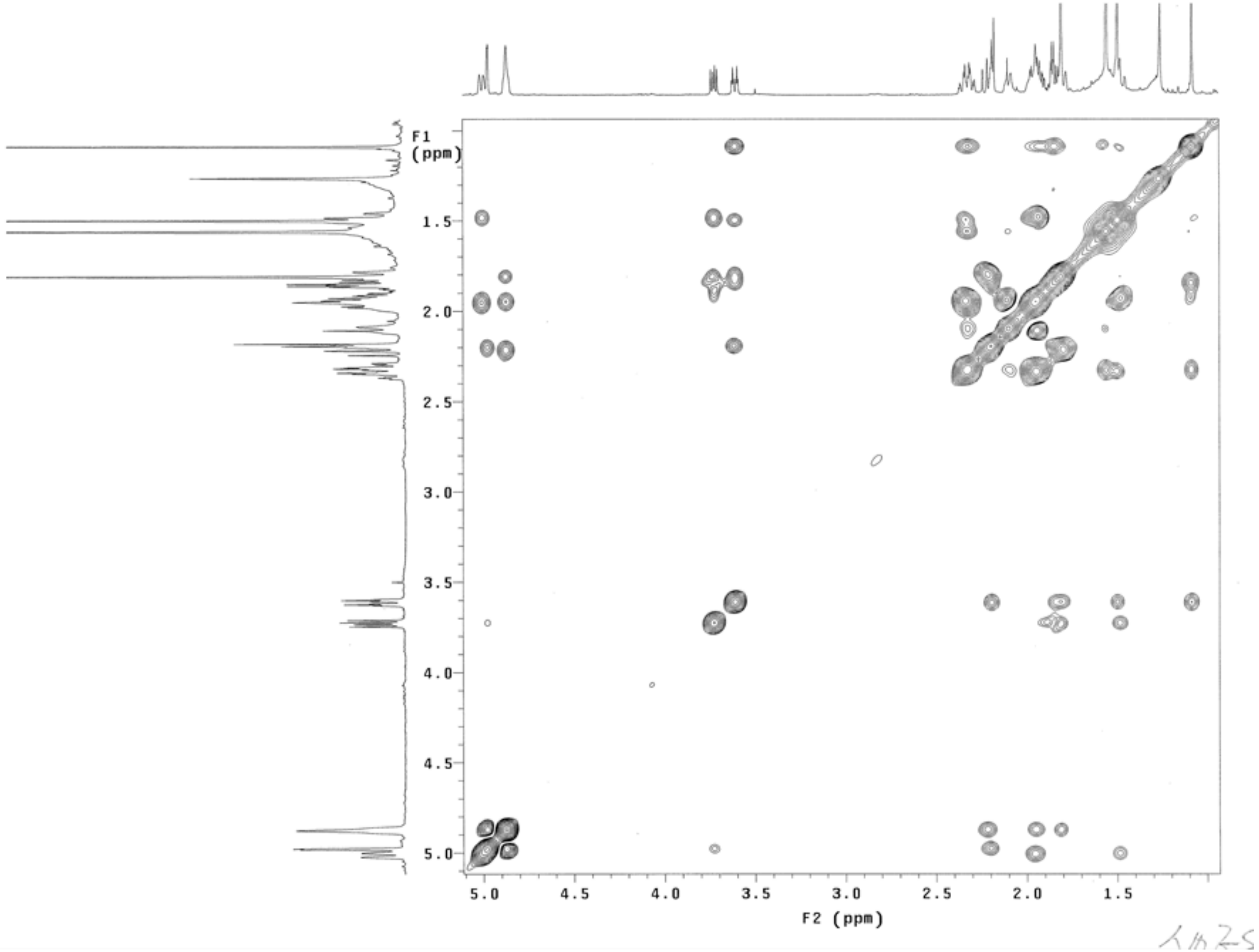

Figure S14: NOESY NMR (500 MHz, $CDCl_3$) spectrum of **2**.

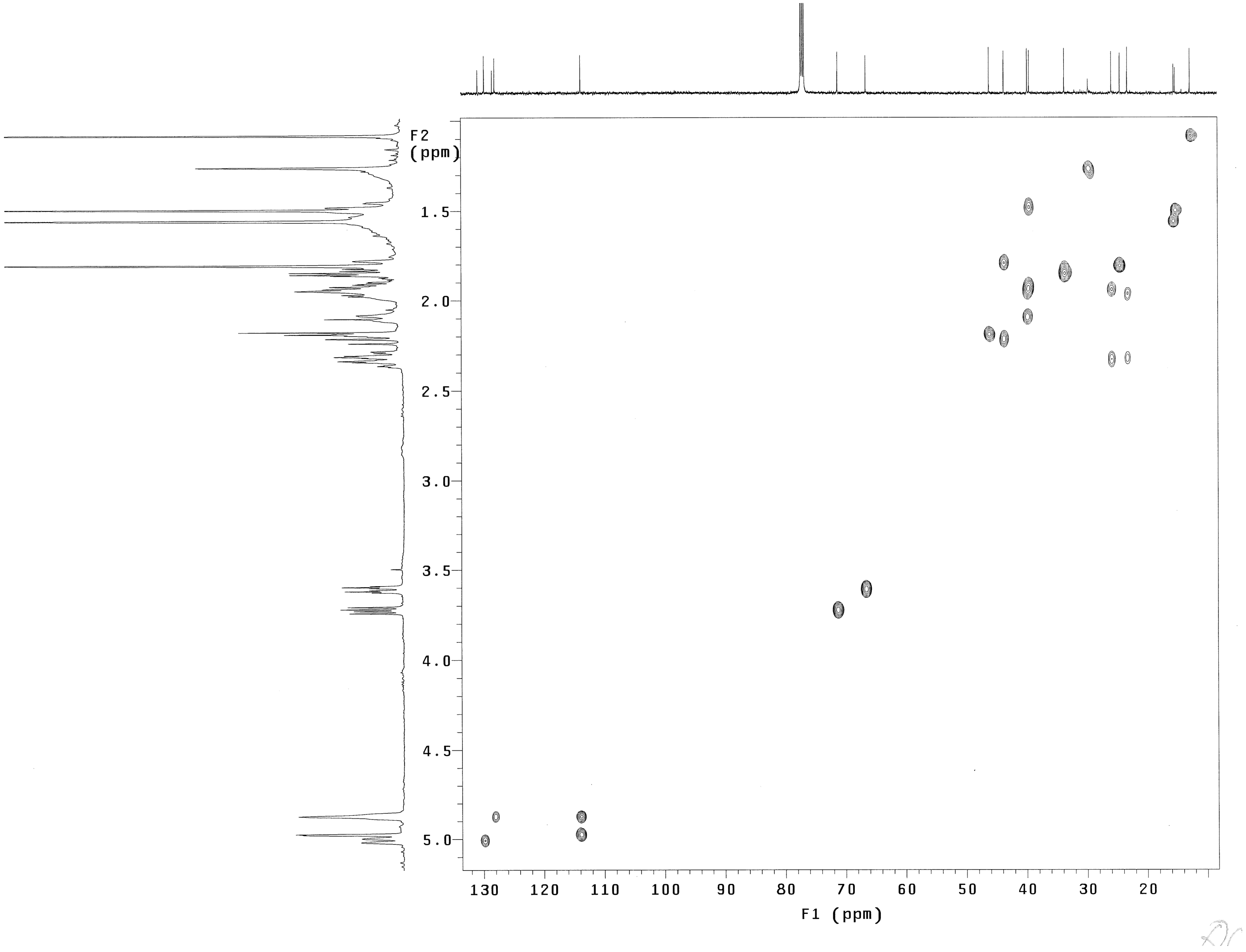

Figure S15: HSQC NMR (500 MHz, $CDCl_3$) spectrum of **2**.

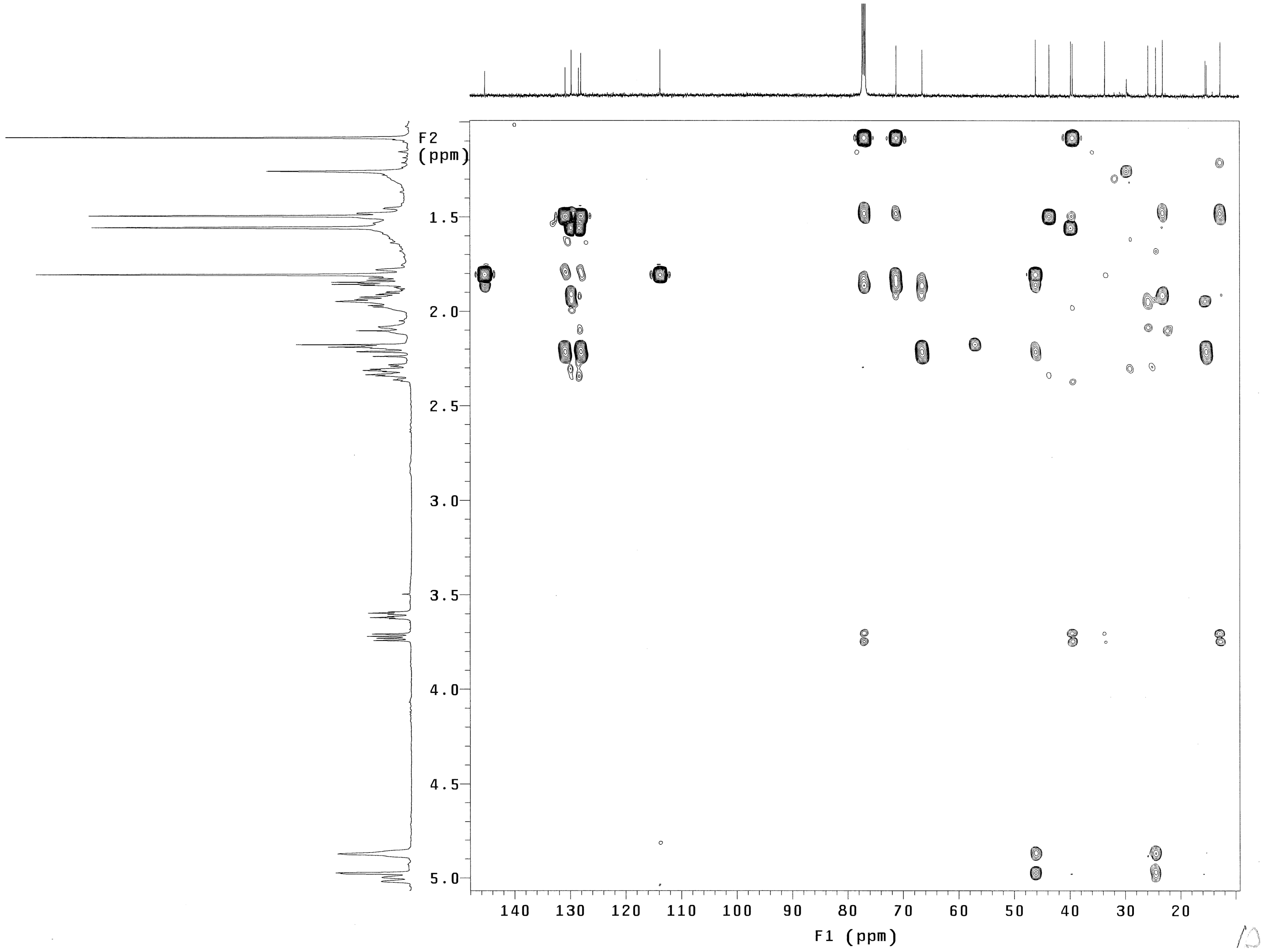

Figure S16: HMBC NMR (500 MHz, $CDCl_3$) spectrum of **2**.

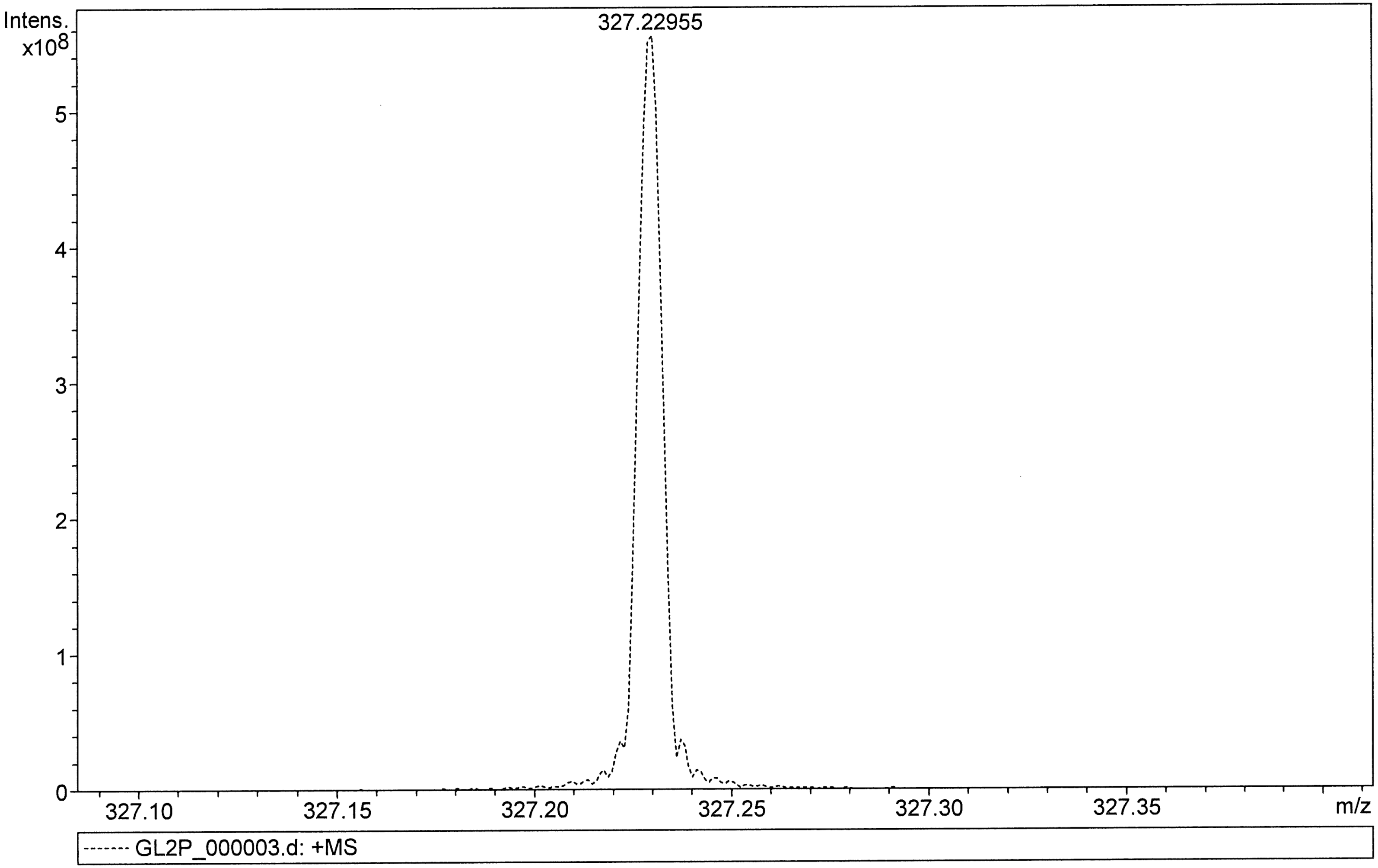

Meas. m/z	#	Formula	Score	m/z	err [mDa]	err [ppm]	mSigma	rdb	e⁻ Conf	N-Rule
327.22955	1	C 20 H 32 Na O 2	100.00	327.22945	-0.10	-0.30	4.3	4.5	even	ok

Figure S17: HRESIMS spectrum of **2**.

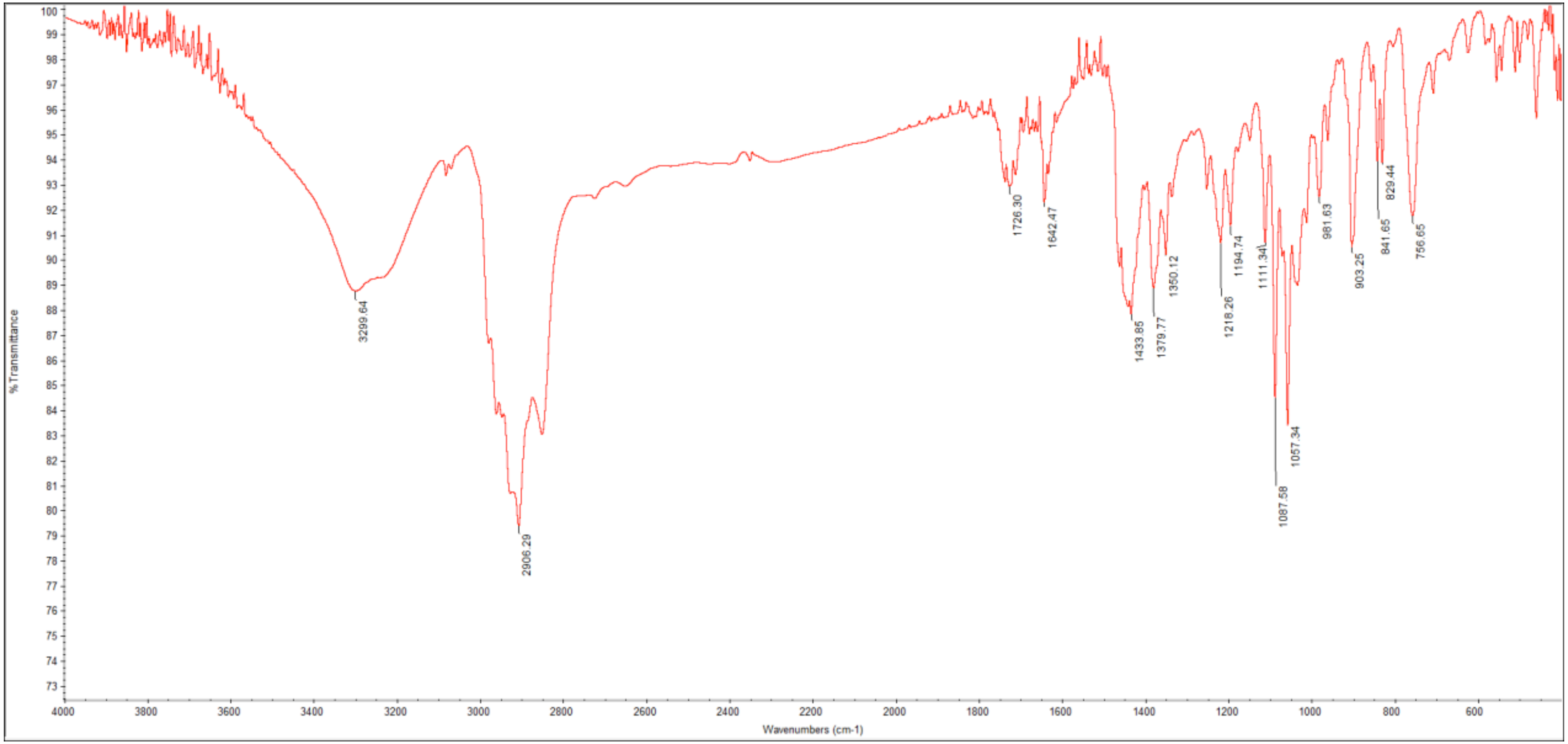

Figure S18: IR spectrum of **2**.

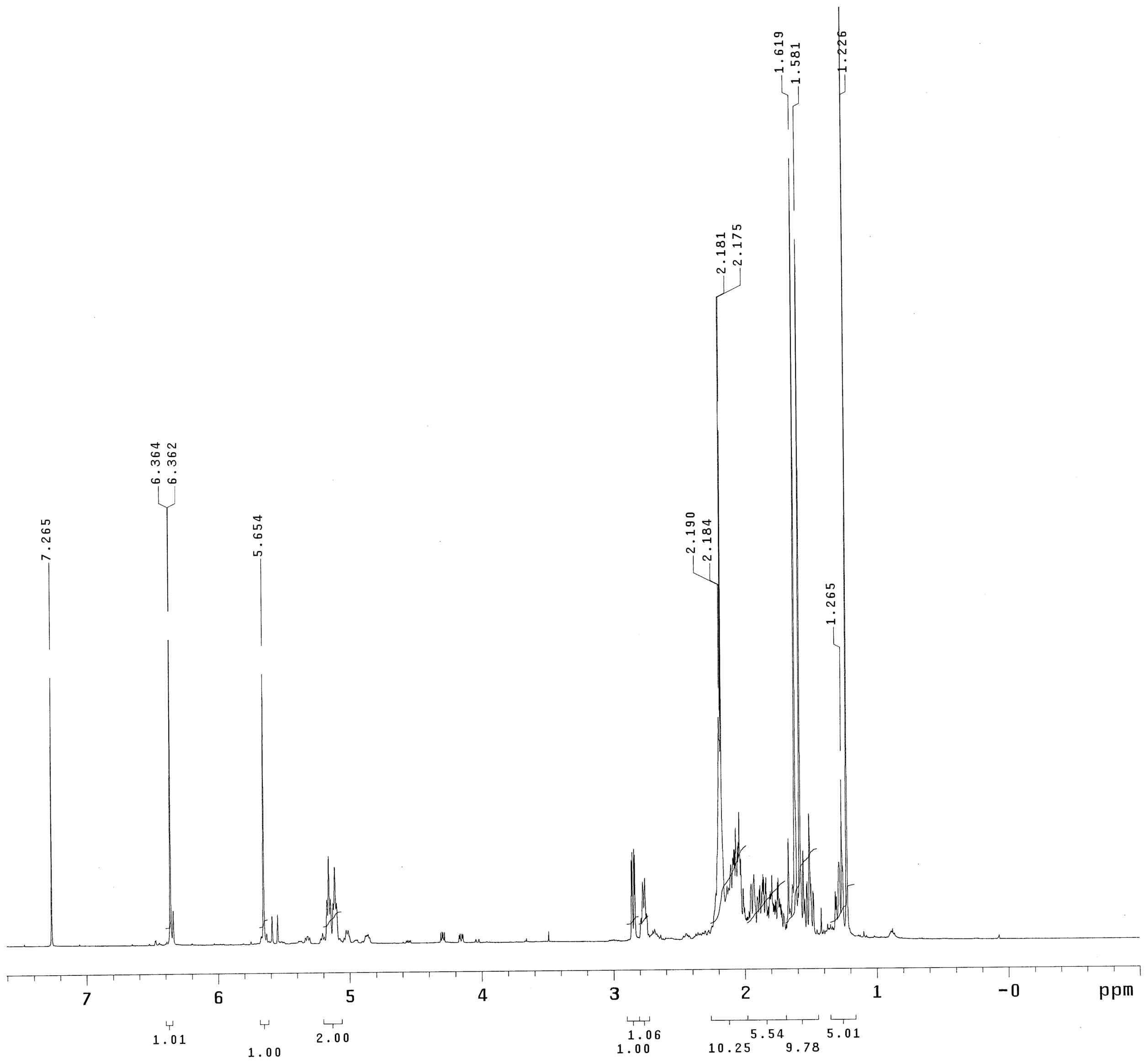

Figure S19: ^{1}H NMR (500 MHz, $CDCl_3$) spectrum of **3**.

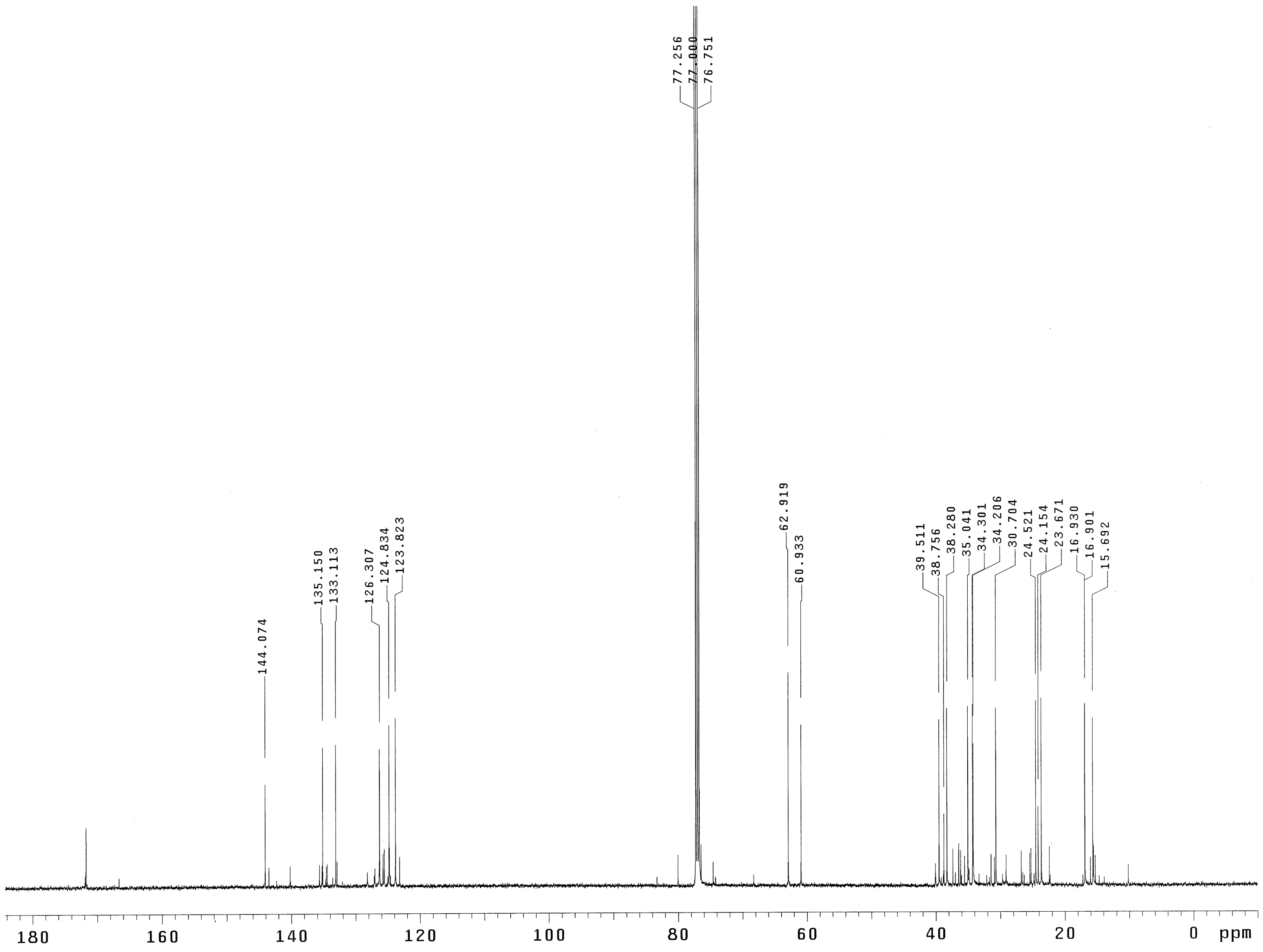

Figure S20: ^{13}C NMR (125 MHz, $CDCl_3$) spectrum of **3**.

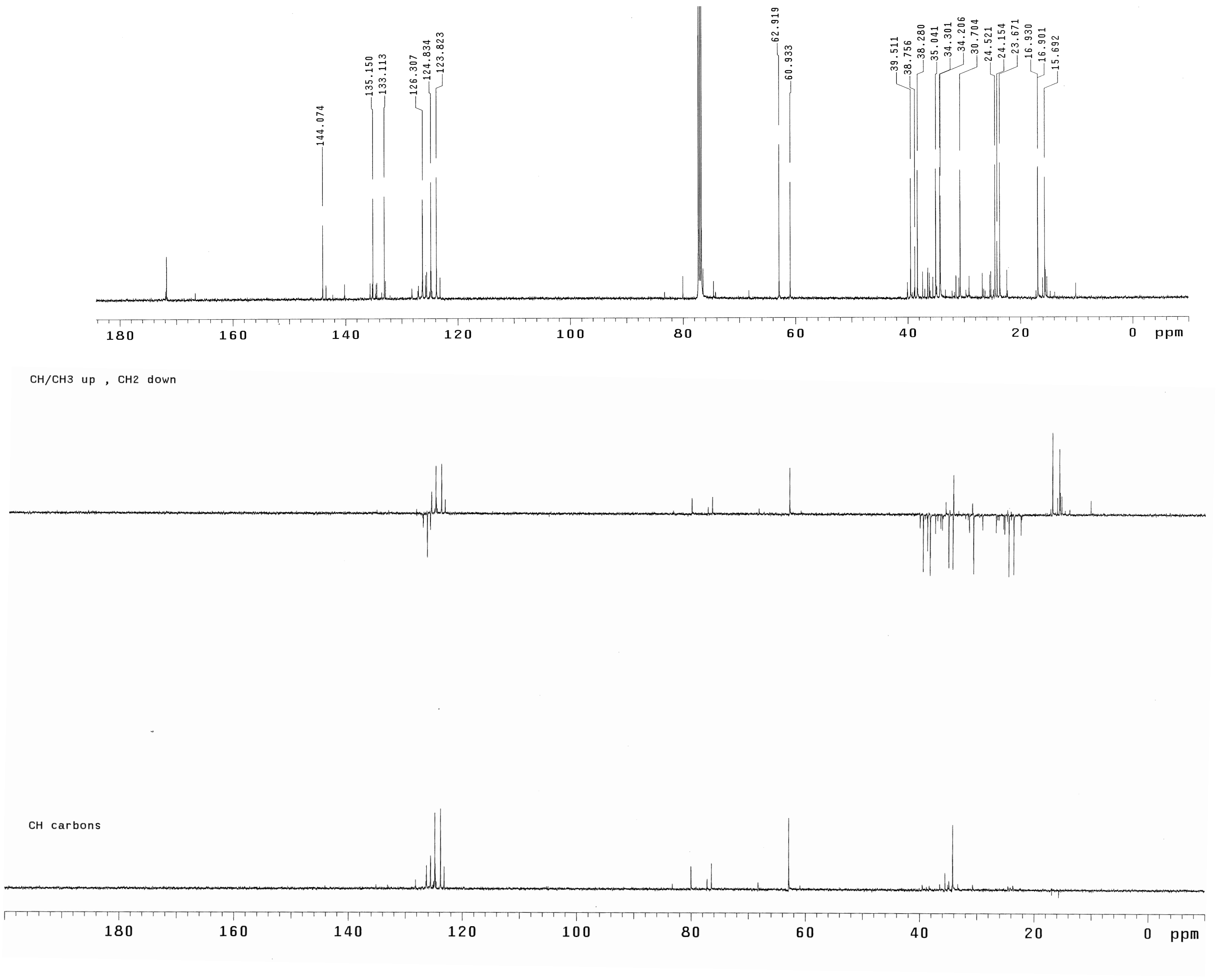

Figure S21: DEPT NMR (125 MHz, $CDCl_3$) spectrum of **3**.

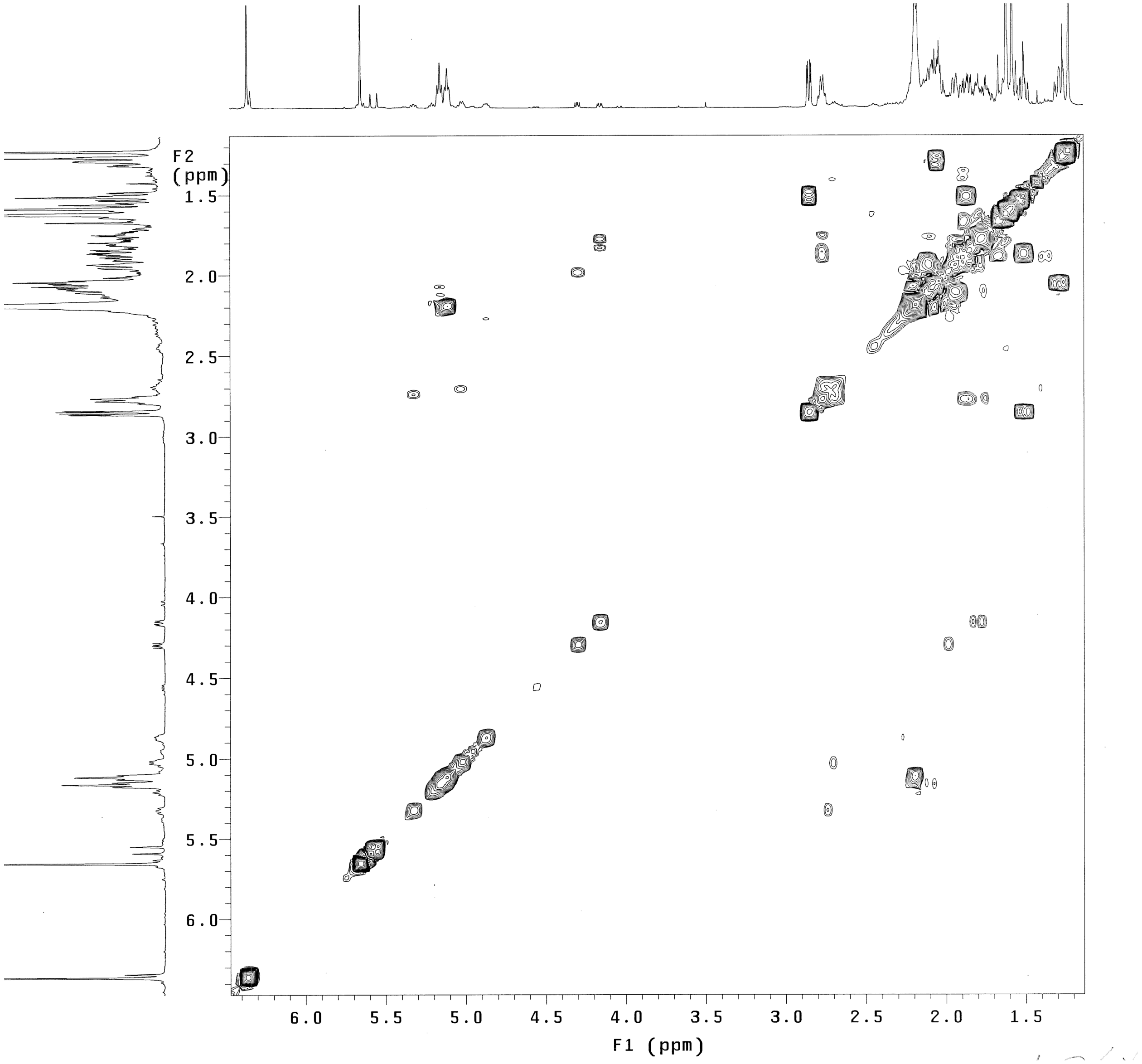

Figure S22: COSY NMR (500 MHz, $CDCl_3$) spectrum of **3**.

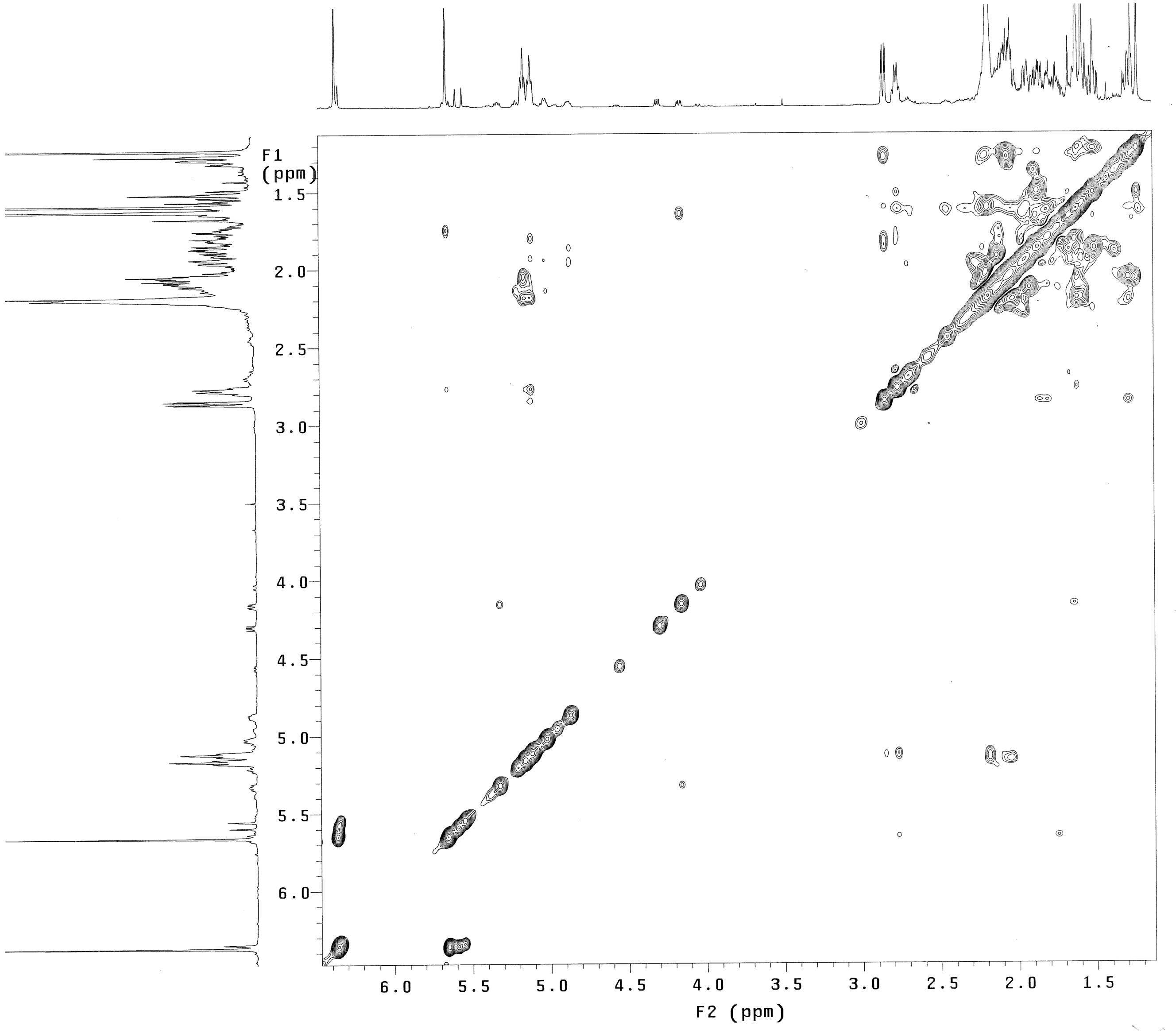

Figure S23: NOESY NMR (500 MHz, $CDCl_3$) spectrum of **3**.

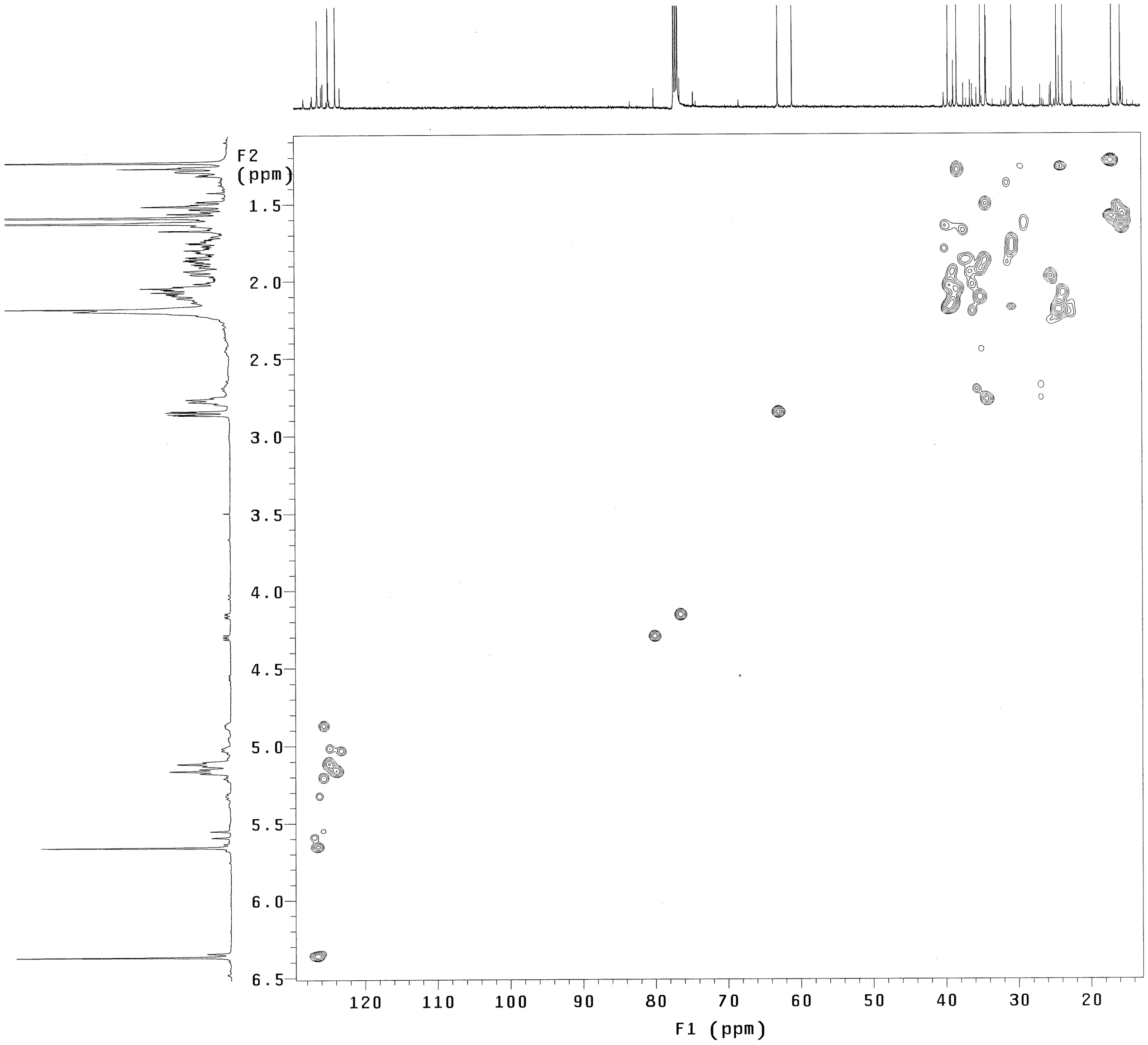

Figure S24: HSQC NMR (500 MHz, $CDCl_3$) spectrum of **3**.

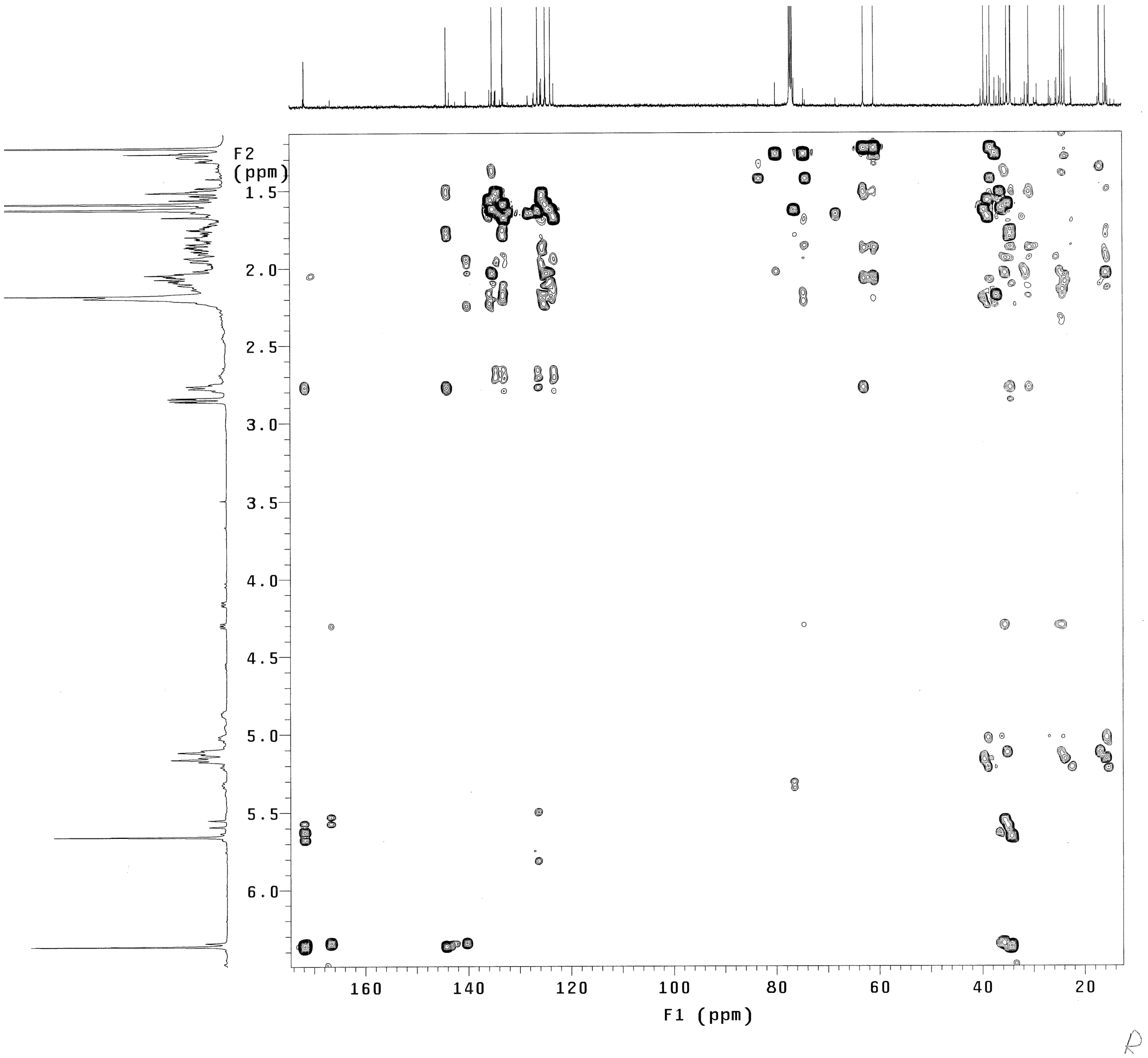

Figure S25: HMBC NMR (500 MHz, $CDCl_3$) spectrum of **3**.

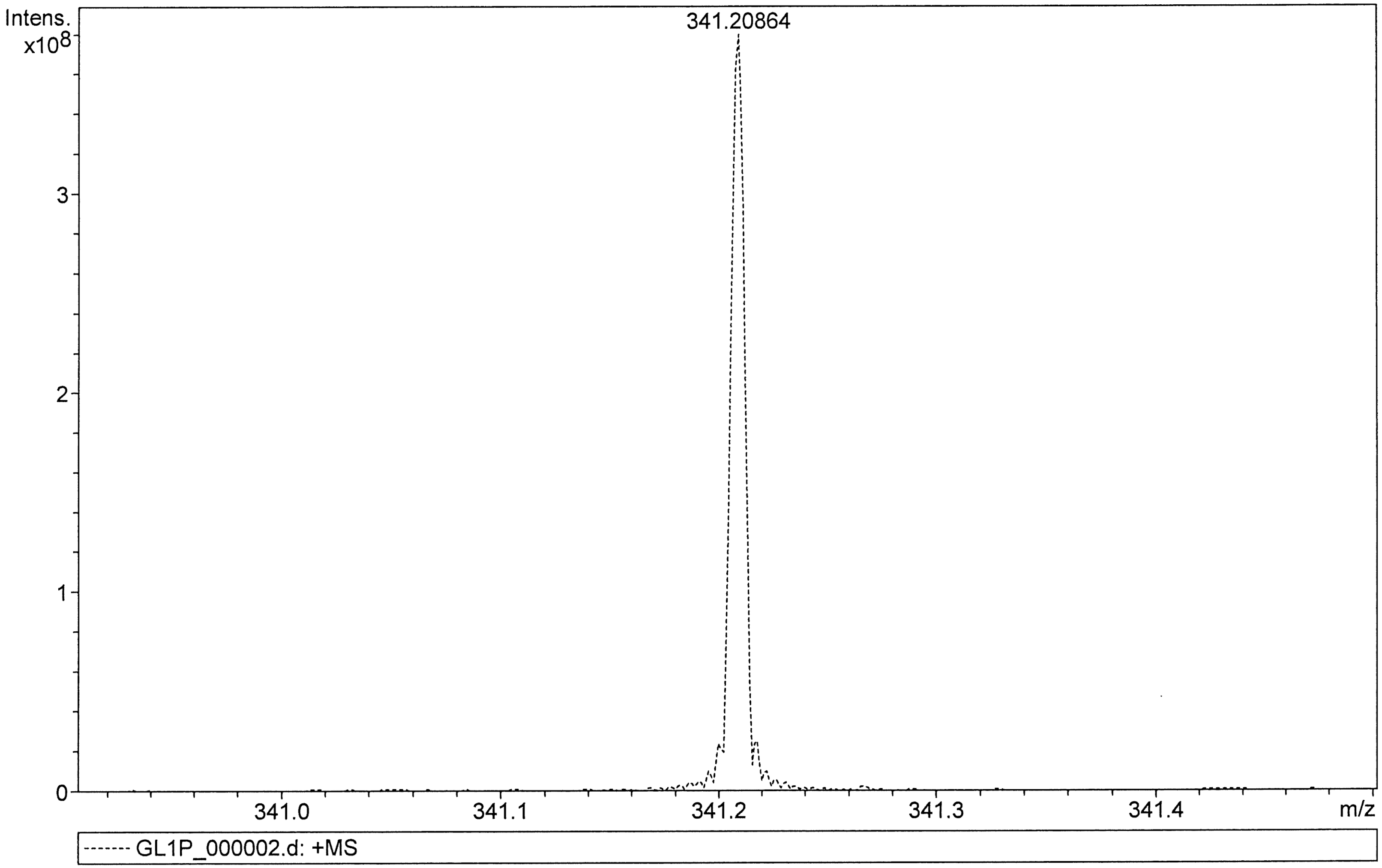

Meas. m/z	#	Formula	Score	m/z	err [mDa]	err [ppm]	mSigma	rdb	e⁻ Conf	N-Rule
341.20864	1	C 20 H 30 Na O 3	100.00	341.20872	0.07	0.22	5.2	5.5	even	ok

Figure S26: HRESIMS spectrum of **3**.

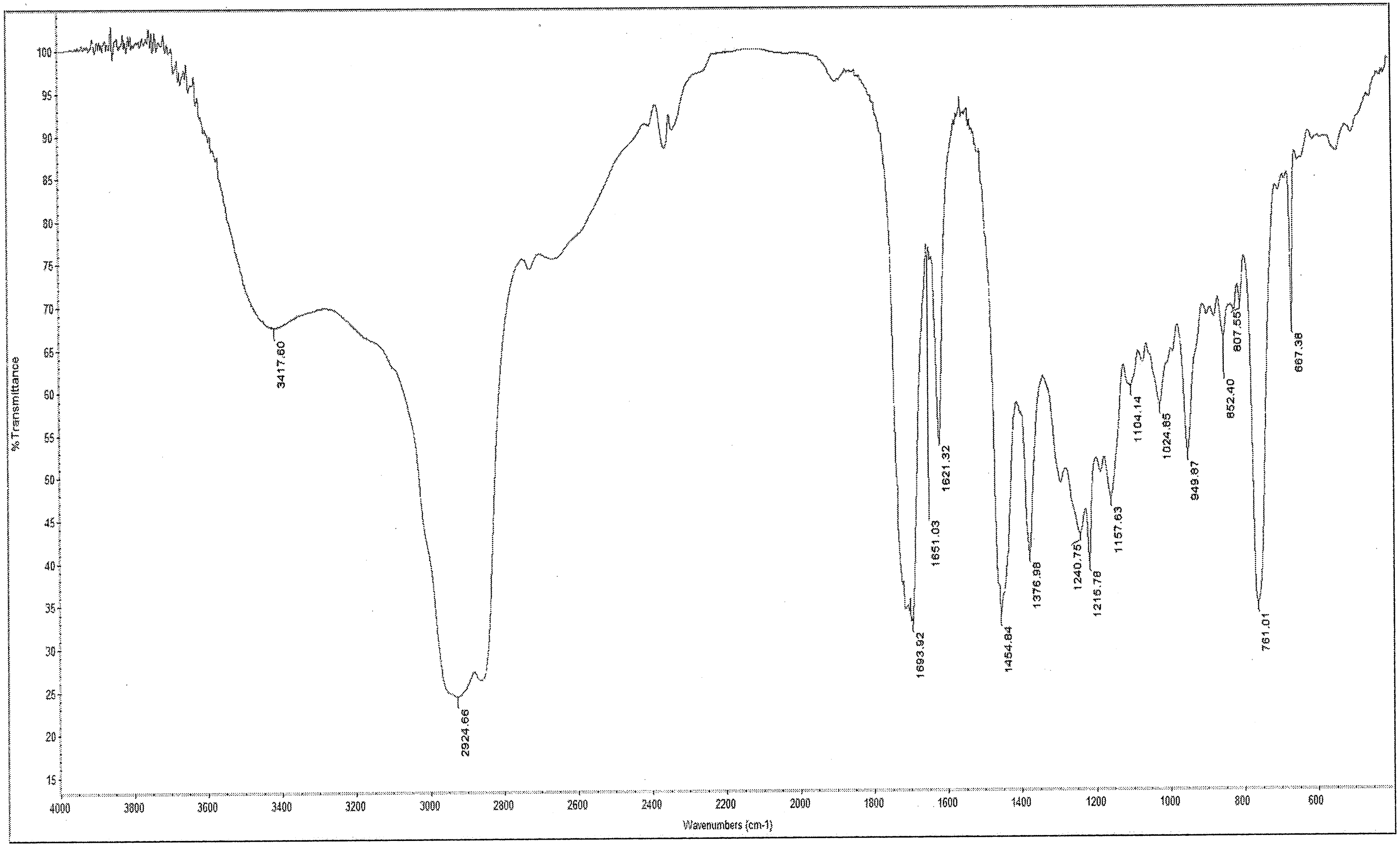

Figure S27: IR spectrum of **3**.
